# Overcoming the Drawbacks of Sulpiride by Means of New Crystal Forms

**DOI:** 10.3390/pharmaceutics14091754

**Published:** 2022-08-23

**Authors:** Rebecca Birolo, Federica Bravetti, Simone Bordignon, Ilenia D’Abbrunzo, Paolo P. Mazzeo, Beatrice Perissutti, Alessia Bacchi, Michele R. Chierotti, Roberto Gobetto

**Affiliations:** 1 Department of Chemistry, University of Torino, 10125 Torino, Italy; 2 Department of Chemical and Pharmaceutical Sciences, University of Trieste, 34127 Trieste, Italy; 3 Department of Chemical, Life and Environmental Sustainability Sciences, University of Parma, 43124 Parma, Italy

**Keywords:** sulpiride, improved solubility, crystal engineering, solid-state NMR, X-ray diffraction, molecular salts, cocrystals, salt cocrystals, dissolution tests

## Abstract

This study aims at developing new multicomponent crystal forms of sulpiride, an antipsychotic drug. The main goal was to improve its solubility since it belongs to class IV of the BCS. Nine new adducts were obtained by combining the active pharmaceutical ingredient with acid coformers: a salt cocrystal and eight molecular salts. In addition, three novel co-drugs, of which two are molecular salts and one is a cocrystal, were also achieved. All samples were characterized in the solid state by complementary techniques (i.e., infrared spectroscopy, powder X-ray diffraction and solid-state NMR). For systems for which it was possible to obtain good-quality single crystals, the structure was solved by single crystal X-ray diffraction (SCXRD). SCXRD combined with solid-state NMR were used to evaluate the ionic or neutral character of the adducts. In vitro dissolution tests of the new crystal forms were performed and all the adducts display remarkable dissolution properties with respect to pure sulpiride.

## 1. Introduction

**Scheme 1:**
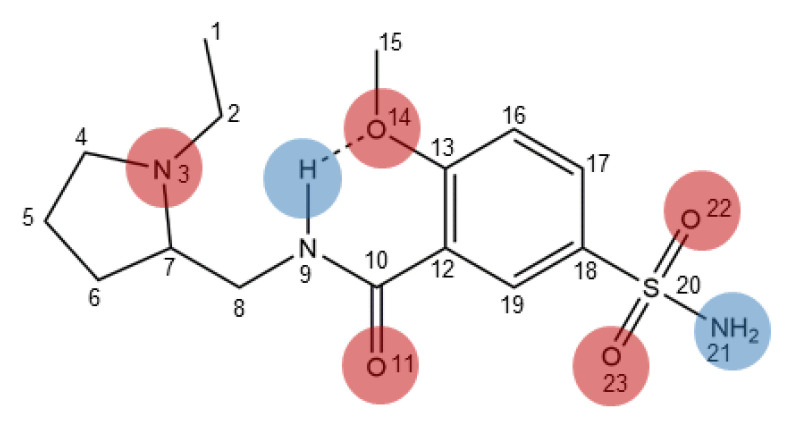
Chemical structure of SULP with atom numbering. The main functional groups available for supramolecular interactions are highlighted in red (H-bond acceptors) and blue (H-bond donors).

Depression and schizophrenia are two major psychotic disorders that interfere with the normal daily life of affected people. The World Health Organization (WHO) data reported that more than 280 million people suffer from depression and 60 million are affected by schizophrenia. Among all antipsychotic drugs, sulpiride (SULP, Scheme 1) is a selective antagonist of dopamine D2 receptors [1]. SULP belongs to the substituted benzamide class, and it is used for the treatment of several psychiatric disorders. Specifically, it is administered orally with the dosage of 200 and 400 mg twice a day as an antipsychotic for schizophrenia and in major depressive disorder, or at a lower dosage of 50 mg three times a day for the treatment of anxiety, gastric and duodenal ulcers, or mild depressive disorders. In the clinical field, SULP has created considerable interest compared to other antidepressant drugs due to numerous advantages, such as low toxicity, fewer extrapyramidal side effects, and a lower affinity for other neuronal receptors, as well as its low cost [2]. However, according to the Biopharmaceutical Classification System (BCS), SULP is classified as class IV because it exhibits low water solubility and limited intestinal permeability [3]. As a result, the drug is slowly and poorly absorbed in the gastrointestinal tract after oral administration. The bioavailability of the drug is approximately 30% and the half-life time is relatively short (6–8 h) [4]. These properties give rise to a high dosage intake, which generally leads to low patient tolerability. Despite these important limitations, SULP remains an effective antipsychotic, thus it is worth developing new strategies to improve its pharmacokinetics. Several drug delivery strategies were developed to enhance its bioavailability, among them microemulsion for nasal administration, microcapsules, solid dispersions, and nanoliposheres were proposed [5,6,7,8]. Instead, a completely different approach was followed by us, since cocrystallization and salification offer a promising chance to improve its performance. It is well known that for most active pharmaceutical ingredients (APIs) there are numerous possible solid forms, such as crystalline or amorphous, single- or multicomponent forms (i.e., polymorphs, salts, cocrystals, hydrates, and solvates), and solid solutions, and each of these has unique physicochemical properties [9]. If the interacting molecules have acidic or basic functional groups, it is possible to observe protonic transfer between the API and the other molecule, called coformer, with formation of a salt [10]. On the other hand, non-ionizable APIs with hydrogen bond (H-bond) donor or acceptor moieties can afford a cocrystal due to the formation of neutral supramolecular synthons [11]. The probability of forming salts or cocrystals is assessable with high success rates through the empirical rule of ∆p*K*_a_ or related models [12], although an experimental assessment of the reaction outcome is always necessary [13,14]. Cocrystallization and salification are among the most effective pharmaceutical development strategies to overcome poor performances of APIs, such as the low aqueous solubility [15]. Other advantages of these crystal forms are the improvement of in vivo pharmacological activity, stability, mechanical properties, and bioavailability, without changing the chemical structure of the APIs. The interest in multicomponent crystal forms also stems from the advantage of being patentable as new solid forms [16]. This kind of approach has been massively used in recent years, as evidenced by the number of publications and the reintroduction into the market of some drugs in the form of salts or cocrystals [17].

To the best of our knowledge, no polymorphs nor multicomponent crystal forms of SULP have been discovered to date. On the Cambridge Structural Database (CSD) only the structures of the hydrochloride salt, of the racemate and of the S-enantiomer of SULP are reported. Considering the chemical structure of SULP, four different functional groups are available for supramolecular interactions (Scheme 1) to design new crystal forms: the tertiary amine, the ether, the amide, and the sulfonamide. In pure SULP, the ether and the amide groups form an intramolecular interaction (i.e., N-H···O), involving a six-term cycle (S(6) motif); therefore, these two groups are not easily available for further supramolecular interactions. Finally, the molecule has five acceptor sites, three H-bond donors, and a basic group, N3 in Scheme 1, with p*K*_a_ = 9.5. For supramolecular syntheses the racemate form of SULP was used.

**Scheme 2:**
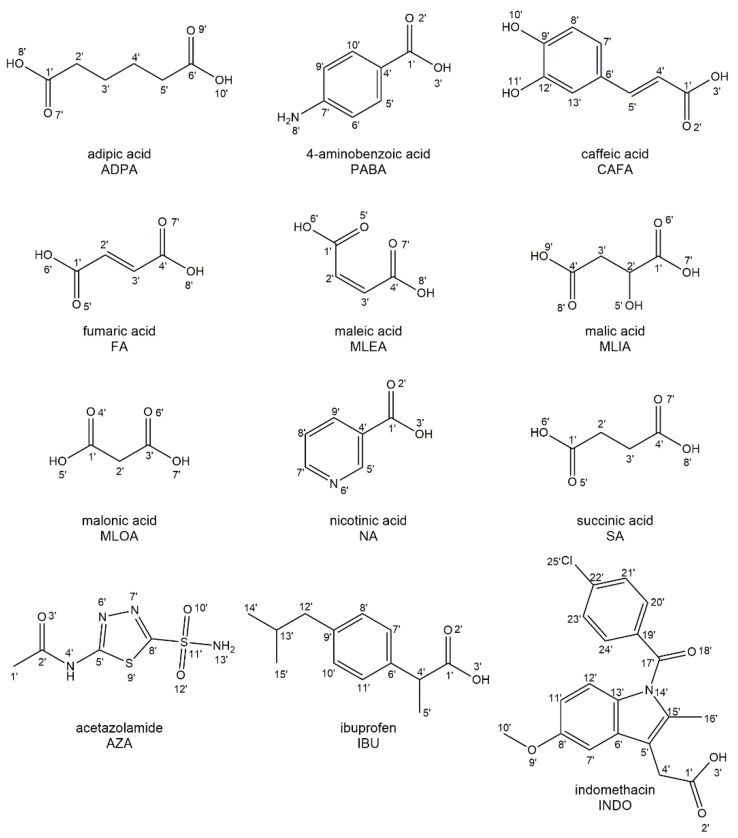
Chemical structure of employed coformers with atom numbering.

The coformers chosen for the design of the adducts are mainly GRAS [18] (Generally Recognized As Safe) molecules widely employed in both food and pharmaceuticals, and often introduced in drug formulations as excipients. Specifically, the coformers are adipic acid (ADPA), 4-aminobenzoic acid (PABA), caffeic acid (CAFA), fumaric acid (FA), maleic acid (MLEA), malic acid (MLIA), malonic acid (MLOA), nicotinic acid (NA), succinic acid (SA) and the active ingredients are acetazolamide (AZA), ibuprofen (IBU), and indomethacin (IND), see Scheme 2. Among the selected coformers, some are of particular interest not only for the possibility of forming a supramolecular adduct but also for their health benefits [19]. For example, NA is a member of the water-soluble B-vitamin family, and its use results in the reduction of the number of blood lipids and in the risk of myocardial infarctions [20]. Differently, SA is administered as a dietary supplement, particularly for the treatment of dietary deficiencies and imbalances, and CAFA has antioxidant properties that protect against free radical-induced DNA damage [21]. Further, several selected coformers are currently used as pharmaceutical excipients (e.g., ADPA, MLIA, FA, and MLEA), mainly as acidifying, buffering, and flavoring agents.

Twelve new adducts were obtained by the slurry technique (see Materials and Methods) and completely characterized by infrared spectroscopy (FTIR-ATR) [22], powder X-ray diffraction (PXRD), ^13^C and ^15^N solid-state NMR (SSNMR), DFT optimization, and SSNMR calculation that allowed discrimination between salts and cocrystals. For systems for which it was possible to obtain good-quality single crystals, i.e., **SULP-ADPA**, **SULP-FA**, **SULP-MLEA**, **SULP-MLOA**, **SULP-IND**, and **SULP-AZA**, the crystal structure was solved by single crystal X-ray diffraction (SCXRD). Finally, in vitro dissolution tests of the new crystal forms were performed to assess whether the adducts performed better than SULP.

## 2. Materials and Methods

**Materials.** SULP (N-[(1-ethylpyrrolidin-2-yl)methyl]-2-methoxy-5-sulfamoylbenzamide) was purchased from Tokyo Chemical Industry (TCI, Milan, Italy) with a declared purity > 98% and used for the preparation of adducts without further purification. ADPA, FA, MLEA, MLIA, MLOA, NA, and SA coformers were purchased from Sigma Aldrich. IBU and AZA were purchased from Alfa Aesar; CAFA from TCI; and PABA from Riedel-deHaën.

**Preparation techniques.** All salts and cocrystals were obtained by means of the slurry technique [23]. In all cases, 100 mg of raceme SULP was mixed with each of the twelve selected coformers, using the appropriate stoichiometric ratio (see Supplementary Materials, Table S1). Then, 1 mL of a solvent chosen from ethanol, methanol, or acetonitrile, depending on the system (see Supplementary Materials, Table S1), was added and left under agitation for 2 days. Once dried, the powdered samples were analyzed and characterized by FTIR-ATR, PXRD and SSNMR. For **SULP-ADPA**, **SULP-FA**, **SULP-MLEA**, **SULP-MLOA**, **SULP-IND,** and **SULP-AZA** adducts it was possible to grow a single crystal of appropriate size for SCXRD analysis by the seeding technique. In separate vials, SULP (20 mg) and 1 or 2 equivalents of the respective coformer, depending on the stoichiometry of the adduct (see Supplementary Materials, Table S1), were dissolved in a minimum amount of solvent. The solvent used for the crystallization of the adducts was ethanol, except for **SULP-MLEA** for which acetonitrile was added. Dissolution was aided by heating, then a spatula tip of the adduct previously synthesized via slurry was added as a nucleation seed. Then, the solutions were left to slowly evaporate at room temperature.

**IR Spectroscopy.** Fourier transform infrared (FT-IR) spectra were recorded on an Equinox 55 (Bruker, Milan, Italy) spectrometer with an ATR reflectance attachment. Spectra were collected in the 400–4000 cm^−1^ range with a resolution of 2 cm^−1^ and 16 scans.

**X-ray Powder Diffraction.** X-ray powder patterns of samples obtained by slurry were recorded on an Xpert Pro (45 kV, 40,000 μA) diffractometer in the Bragg–Brentano geometry, using Cu−Kα radiation (λ = 1.5418 Å) in the 2θ range between 5° and 50° (continuous scan mode, step size 0.0167°, counting time 40 s).

**Solid-State NMR.** Solid-state NMR spectra were acquired with a Bruker Avance II 400 Ultra Shield instrument, operating at 400.23, 100.63, and 40.56 MHz, respectively, for ^1^H, ^13^C, and ^15^N nuclei. The powdered samples were packed into cylindrical zirconia rotors with a 4 mm o.d. and an 80 µL volume. A certain amount of sample was collected from the batch and used without further preparation to fill the rotor. ^13^C CPMAS spectra were acquired at a spinning speed of 12 kHz, using a ramp cross-polarization pulse sequence with a 90° ^1^H pulse of 3.60 µs, a contact time of 3 ms, optimized recycle delays between 3 and 15 s, and a number of scans in the range 200–1000, depending on the sample. ^15^N CPMAS spectra were acquired at a spinning speed of 9 kHz using a ramp cross-polarization pulse sequence with a 90° ^1^H pulse of 3.60 µs, a contact time of 4 ms, optimized recycle delays between 3 and 15 s, and a number of scans in the range 20,000–50,000, depending on the sample. For all spectra, a two-pulse phase modulation (TPPM) decoupling scheme was used, with a radiofrequency field of 69.4 kHz. The ^13^C and ^15^N chemical shift scales were calibrated through the signals of γ-glycine (^13^C methylenic peak at 43.7 ppm and ^15^N peak at 33.4 ppm with reference to NH_3_).

**Single Crystal X-ray Diffraction.** SCXRD analysis was performed on selected single crystal samples of **SULP-ADPA**, **SULP-FA**, **SULP-MLEA**, **SULP-MLOA**, **SULP-IND,** and **SULP-AZA** on a Bruker D8 Venture diffractometer equipped with a kappa goniometer and a Photon II detector. Low temperature data collections (200 K) were performed under nitrogen flux by means of an Oxford cryostream. Microfocused Cu−Kα radiation (λ = 1.541846 Å) was used for **SULP-ADPA**, **SULP-FA**, **SULP-MLOA,** and **SULP-AZA,** while microfocused Mo−Kα radiation (λ = 0.71073 Å) was used for **SULP-MLEA** and **SULP-IND**; Lorentz polarization and absorption correction were applied. Data were post-processed using APEX3 Crystallography Software Suite (Bruker AXS Inc., Madison, Wisconsin, USA). Structures were solved by direct methods using SHELXT [24] and refined by full-matrix least-squares on all F^2^ using SHELXL [25] implemented in Olex2.15 [26]. Anisotropic displacement parameters were refined except for hydrogen atoms. **SULP-FA** and **SULP-IND** crystals were poorly diffracting at higher 2θ values with I/σ > 3 below the limit acknowledged by the IUCr. This returned quite a high R_1_ value for these two structures. **SULP-AZA** was instead refined as twinned crystal. Table 1 reports crystal data collection and refinement results. ORTEP diagrams are reported in Supplementary Materials. A table with all the H-bond interactions can be found in the Supplementary Materials (Table S3). Crystallographic data for all structures have been deposited at the Cambridge Crystallographic Data Centre under deposition number CCDC 2175373-2175378 er. Copies of these data can be obtained free of charge via www.ccdc.cam.ac.uk/data_request/cif (accessed on 8 July 2022) or by emailing data_request@ccdc.cam.ac.uk, or by contacting The Cambridge Crystallographic Data Centre, 12 Union Road, Cambridge CB2 1EZ, UK; Fax: +44-1223-336033.

**In vitro dissolution studies**. Prior to dissolution, solubility measurements of raw SULP were carried by adding an excess amount of the drug to 10 mL of distilled water. The suspensions were kept under agitation in the dark, at 37 °C for 70 h, to ensure that equilibrium was attained, and then filtered through a membrane filter (pore size 0.22 µm). The value of 1.1930 ± 0.1297 g/L (mean ± S.D, *n* = 3) as SULP water solubility at 37 °C was obtained, in agreement to literature data [27]. For the in vitro dissolution test, a dissolution apparatus (Hanson SRplus Dissolution Test Station, Chatsworth, Los Angeles, CA, USA), equipped with a paddle rotating at 100 rpm, was employed. An accurately weighed sample of adduct as a powder (100–50 µm particle diameter class), containing a suitable amount (72.19 mg) of SULP for sink conditions (C < 0.2 C_s_) was dissolved into 950 mL of freshly distilled water, maintained at 37 ± 0.1 °C as dissolution medium. The aqueous solution was filtered and continuously pumped with a peristaltic pump (Mod.SP 311 Velp Scientifica, Milano, Italy) to a flow cell in a spectrophotometer (Biochrom Libra S12, Biochrom, Cambridge, UK) and absorbance values were recorded at the maximum wavelength of the drug (291 nm). The coformers did not interfere with the UV analysis, with the exception of INDO, AZA, CAFA, and PABA, laying on the same wavelength range of SULP. The results were the average of triplicate experiments, and standard deviations did not exceed 5% of mean value. The same procedure was followed for samples of pure drug, as a powder (after selection of the same particle diameter class).

**DFT optimization and Solid-State NMR calculation.** Experimental crystal structures of SULP adducts and pure SULP were fully optimized at the DFT level using Quantum Espresso [28] (v. 6.4.1), employing the nonlocal vdW-DF2 [29] method and the PBE [30,31] pseudopotentials, with an energy cut-off of 60 Ry [32]. Optimized structures were visualized and compared to the experimental one using the Mercury utility “crystal packing similarity”. The root-mean square deviation of the overlay of the experimental and optimized structures with a cluster of 20 molecules (RMSD_20_) is shown in Table S2 in the Supplementary Materials. The solid-state NMR calculation was performed using the Gauge Including Projected Augmented Wave (GIPAW) [33] with an energy cut-off of 80 Ry. The obtained absolute isotropic magnetic shielding (σ_iso_) values were converted into isotropic chemical shifts (δ_iso_) relative to the absolute magnetic shielding of DABCO and used as reference substance, applying Equation (1).

δ_iso_(calc) = σ_iso_(ref) − σ_iso_(calc) (1)

Experimental chemical shifts, δ_iso_(exp) were plotted against σ_iso_(calc) values, resulting in the reference shielding constants, σ_iso_(ref) [34] values of 120.59 and 212.22 for ^13^C and ^15^N, respectively.

## 3. Results and Discussion

**Figure 1:**
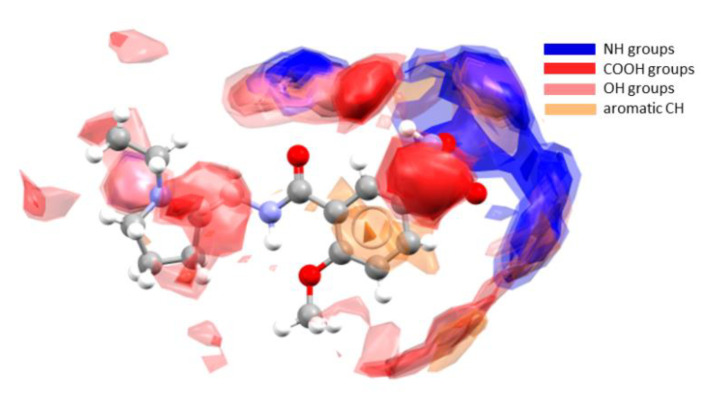
Full interaction map of SULP. Blue color indicates the probability of forming interactions with NH groups, red with COOH groups, pink with OH group, and orange π-π interactions.

The probability of SULP to establish supramolecular interactions with different functional groups was studied by Mercury [35]. N3 (see Scheme 1) tends to interact preferentially with H-bond donor groups, such as OH or COOH; a high probability of forming supramolecular synthons is also related to the sulfonamide group, which may act as both an H-bond donor as well as an acceptor through O and NH groups, respectively (Figure 1).

A new series of crystal forms of SULP were obtained by means of solution cocrystallization techniques, as reported in the Experimental Section. Specifically, three co-drugs (of which two were molecular salts and one a cocrystal), one salt cocrystal [36], and eight molecular salts were obtained. For **SULP-ADPA**, **SULP-FA**, **SULP-MLEA**, **SULP-MLOA**, **SULP-IND,** and **SULP-AZA,** it was possible to solve the structure from SCXRD data. In the case of **SULP-PABA**, **SULP-CAFA**, **SULP-MLIA**, **SULP-NA**, **SULP-SA,** and **SULP-IBU** for which no X-ray structure was available, the structural characterization relied on complementary spectroscopic analyses only. FTIR-ATR spectra (Figures S1–S12 in the Supplementary Materials) and X-ray powder diffractograms (Figures S13–S24 in the Supplementary Materials) were used mainly to identify the formation of new crystalline phases. The ionic character of all adducts was assessed by means of ^13^C and ^15^N CPMAS SSNMR experiments supported by SCXRD, when available. In fact, it is well-known that ^13^C and ^15^N SSNMR represent excellent methods for detecting protonic transfers since these induce relevant modifications in the position of the signals of the involved groups [37]. Focusing on our systems, a carboxylic acid group is present in each coformer: a high-frequency shift of its signal in ^13^C CPMAS spectrum of the adduct indicates a salt formation. Conversely, a low-frequency shift highlights the achievement of a cocrystal. For systems for which the protonation state was ambiguous from ^13^C data, ^15^N CPMAS spectra and ab initio calculations provided unequivocal evidence.

### 3.1. Crystal Structures

**SULP-ADPA**, **SULP-FA**, **SULP-MLEA**, **SULP-MLOA,** and **SULP-AZA** crystallized as anhydrous salts with the exception of **SULP-IND** that crystallized as a monohydrate salt. The position of the hydrogen atom at the pyrrolidinic nitrogen (N3) was experimentally determined. The ether group of SULP is always involved in the formation of the intramolecular S(6) H-bond ring with the close amidic group, which prevents them from being further involved in other intermolecular interactions. Comparisons of diffractograms from experimental powders with those calculated by Mercury from the derived structures are shown in the Supplementary Materials (Figures S25–S30). This allowed us to confirm that the single crystals selected for the analyses were representative of the bulk.

**Figure 2:**
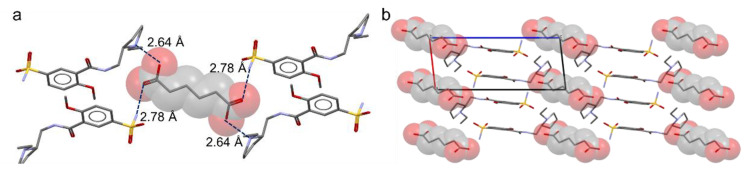
(**a**) Intermolecular interactions established between adipate and surrounding SULP molecules (dashed blue lines). ESDs are omitted for the sake of clarity. (**b**) Crystal packing of **SULP-ADPA** along the (010) direction. Color code C = grey, N = blue, O = red, S = yellow. ADPA ions are highlighted with spacefill style shadow. Hydrogen atoms are omitted for the sake of clarity.

**SULP-ADPA** crystallizes in the $P\bar{1}$ space group in a 2:1 stoichiometric ratio. Each adipate ion bridges four symmetry-related hydrogenated SULP molecules through the pyrrolidine ring (N3···O8*′*/O10*′* = 2.64(2) Å) and the NH_2_ group (N21···O7*′*/O9*′* = 2.78(1) Å) (Figure 2a). The coformers are arranged within the crystal structure in alternated columns that run along the crystallographic *b* axis (Figure 2b). Additionally, the SULP molecules form symmetry-related dimers with the C=O of the amidic group interacting with the amine decoration of the adjacent molecules (N21···O11*′* = 2.89(2) Å), thus forming an $R_{2}^{2}\left(16\right)$ H-bond ring.

**Figure 3:**
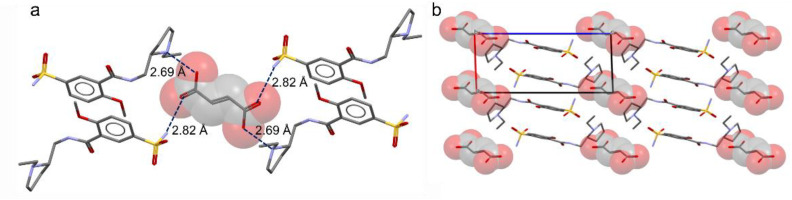
(**a**) Intermolecular interactions established between fumarate and the surrounding hydrogenated SULP molecules (dashed blue lines). ESDs are omitted for the sake of clarity. (**b**) Crystal packing of **SULP-FA** along the (010) direction. Color code C = grey, N = blue, O = red, S = yellow. FA ions are highlighted with spacefill style shadow. Hydrogen atoms are omitted for the sake of clarity.

**SULP-FA** is isostructural with **SULP-ADPA** and crystallized in the space group $P\bar{1}$ in a 2:1 stoichiometric ratio (Figure 3a,b). The resulting interactions are N3···O6*′*/O8*′* = 2.69(2) Å and N21···O5*′*/O7*′* = 2.82(1) Å (Figure 3a). A symmetry-related SULP dimer as observed for **SULP-ADPA** is present (N21···O11*′* = 2.98(2) Å), thus forming an $R_{2}^{2}\left(16\right)$ H-bond ring.

**Figure 4:**
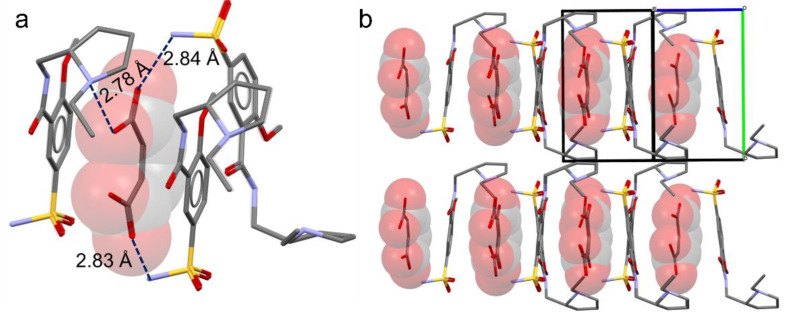
(**a**) Intermolecular interactions established between maleate moiety and surrounding hydrogenated SULP molecules (dashed blue lines). ESDs are omitted for the sake of clarity. (**b**) Crystal packing of **SULP-MLEA** along (101) direction. Color code C = grey, N = blue, O = red, S = yellow. MLEA ions are highlighted with spacefill style shadow. Hydrogen atoms are omitted for the sake of clarity.

**SULP-MLEA** crystallizes in the Pn space group in a 1:1 stoichiometric ratio (Figure 4a,b). Hydrogenated SULP and maleate counterions are arranged in columns that run along the crystallographic *a* axis in an alternated fashion within the crystal structure, as observed in Figure 4b. The N3···O6*′* distance is 2.78(3) Å. Each maleate moiety bridges two additional hydrogenated SULP molecules (Figure 4a) via an H-bond through their NH_2_ group (N21···O7*′* = 2.83(1) Å and N21···O5*′* = 2.84(2) Å, respectively). No significant intermolecular homomeric interactions among SULP molecules are observed.

**Figure 5:**
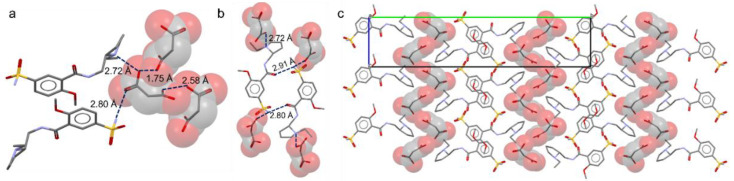
(**a**) Intermolecular interactions established between malonate moiety and surrounding SULP molecules (dashed blue lines). (**b**) Homomeric interactions between SULP dimers are also reported. ESDs are omitted for the sake of clarity. (**c**) Crystal packing of **SULP-MLOA** along (101) direction. Color code C = grey, N = blue, O = red, S = yellow. MLOA ions are highlighted with spacefill style shadow. Hydrogen atoms are omitted for the sake of clarity.

**SULP-MLOA** crystallized in the $P2_{1}/c$ space group in a 1:1 stoichiometric ratio (Figure 5a–c). The SULP molecules form symmetry-related dimers with the C=O of the amidic group interacting with amine decoration of the adjacent molecules (2.91(1) Å), thus forming an $R_{2}^{2}\left(16\right)$ H-bond ring (Figure 5b). Conversely, from what has been observed in the SULP-ADPA and SULP-FA structures, the malonate ion bridged only two hydrogenated SULP molecules, respectively through the pyrrolidine ring (N3···O5*′* = 2.72(2) Å) and the NH_2_ group (N21···O4*′* = 2.80(1) Å) (Figure 5a). Malonate ions form zigzag chains that run along the crystallographic *c* axis. The SULP and malonate counterions are arranged in an alternated fashion within the crystal structure, as observed in Figure 5c.

**Figure 6:**
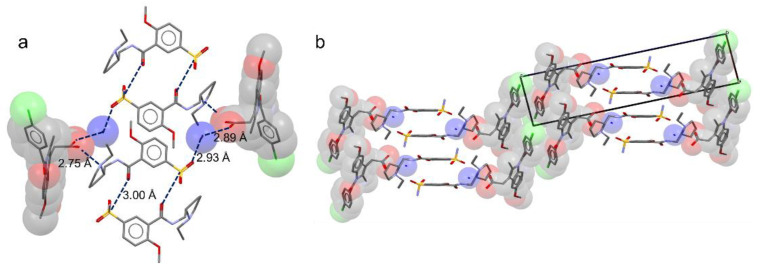
(**a**) Intermolecular interactions established between IND anions and the hydrogenated SULP molecules (dashed blue lines). Homomeric interaction between SULP dimers are also reported. ESDs are omitted for the sake of clarity. (**b**) Crystal packing of **SULP-IND** along (010) direction. Color code C = grey, N = blue, O = red, S = yellow, Cl = green. IND ions are highlighted with spacefill style shadow. Hydration water molecules are highlighted with blue spacefill style shadow. Hydrogen atoms are omitted for the sake of clarity.

**SULP-IND** crystallized in the $P\bar{1}$ space group as a monohydrated salt in a 1:1 stoichiometric ratio. The SULP molecules form symmetry-related dimers with the C=O of the amidic group interacting with the amine decoration of the adjacent molecules (N21···O11*′* 3.00(1) Å), thus forming an $R_{2}^{2}\left(16\right)$ H-bond ring. The pyrrolidinic group link an IND molecule through its carboxylic group (N3···O3*′* = 2.75(2)Å). Additionally, the SULP molecule bridges a water molecule to an adjacent IND molecule (S=O23···O_water_ = 2.93(2) Å; O_water_···C=O2*′* = 2.89(1) Å) (Figure 6a). Coformers are arranged in columns that run along the crystallographic *b* axis (Figure 6b).

**Figure 7:**
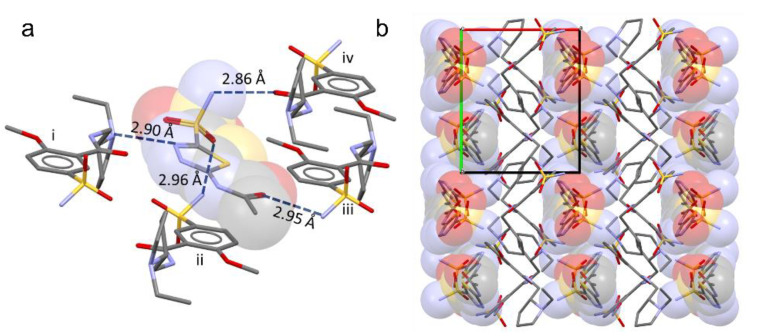
(**a**) Intermolecular interactions established between AZA anion and surrounding hydrogenated SULP molecules (dashed blue lines). ESDs are omitted for the sake of clarity. (**b**) Crystal packing of **SULP-AZA** along (001) direction. Color code C = grey, N = blue, O = red, S = yellow. AZA anions are highlighted with spacefill style shadow. Hydrogen atoms are omitted for the sake of clarity.

**SULP-AZA** crystallized in the $P2_{1}/c$ space group in a 1:1 stoichiometric ratio. AZA is largely decorated with H-bond donor and acceptor groups; thus, it is surrounded by four SULP molecules, labelled from i to iv in Figure 7a. SULP i interacts through its pyrrolidinic ring with the thiadiazole ring of its counterion (N3···N7*′* = 2.90(4) Å). SULP ii and iii both interact with their SNH_2_ group, respectively, with the S=O (N21···O10*′* 2.96(3) Å) and amidic C=O (N21···O3*′* = 2.95(2) Å) groups of the AZA. SULP iv acted as an H-bond acceptor through its C=O amidic group with the SNH_2_ (O11···N13*′* = 2.86(5) Å) of the AZA molecules. An intramolecular S(6) ring is also present in the SULP molecule (N9···O14 = 2.63(4) Å. Coformers are arranged in columns that run along the crystallographic *b* axis (Figure 7b).

### 3.2. Solid-State NMR

**Figure 8:**
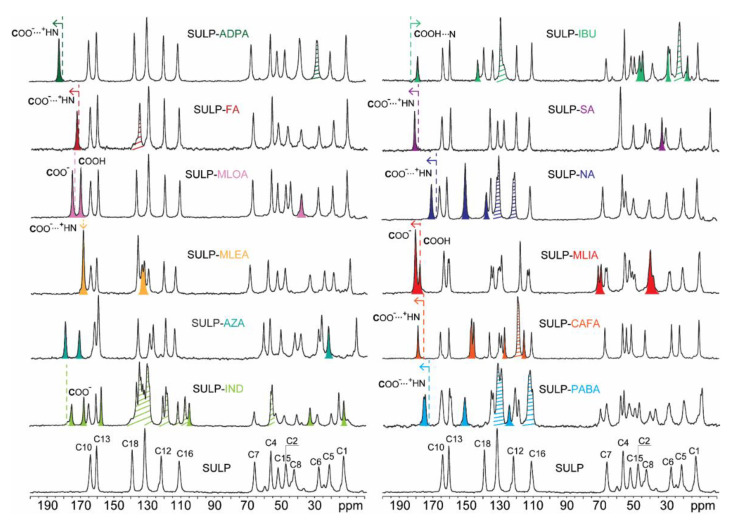
^13^C (100 MHz) CPMAS spectra of SULP (with assignment) and of all the 12 achieved adducts, acquired with a spinning speed of 12 kHz at room temperature. The COOH signals of the pure coformers are represented by the colored dashed lines in each spectrum to highlight the shift undergone upon cocrystallization. Colored peaks represent signals ascribable to the coformer. Striped peaks result from the overlap of coformer signals with those of SULP. The numbering of the SULP atoms refers to Scheme 1.

The ^13^C CPMAS spectra recorded for all of the adducts are shown in Figure 8, where they are compared with that of pure SULP. The ^13^C CPMAS spectra of all coformers are reported in Figure S37 in the Supplementary Materials. The spectra of the adducts all show consistent shifts of carbon atoms associated with both SULP and with their respective coformers, proving the formation of the 12 new crystalline phases that differ from the starting materials. All spectra point out the high degree of crystallinity of the new crystal forms, evidenced by the average full width at half maximum value for the signals, in the range of 80*–*150 Hz. All selected coformers, except AZA and NA, display in their pure form (see Figure S37 in the Supplementary Materials) at least one carboxylic acid group involved in a homodimeric interaction that results in the signal of this group falling at higher chemical shifts, i.e., between 175*–*180 ppm. If the spectra of the adducts show a slight shift toward higher frequencies, the COOH of the coformer is in the carboxylated form since the dimeric COOH groups and the COO^−^ resonate at similar frequencies, as reported in several papers for analogous systems [38]. The ^13^C CPMAS spectra of **SULP-ADPA** and **SULP-FA** are in perfect agreement with the relative reported structures: the number of signals confirms the stoichiometry, while the shift of the carboxylic signal to higher frequencies indicates its deprotonated state, that is, the formation of two new molecular salts. The **SULP-MLOA** spectrum accurately describes the structural features of the sample; in fact, not only does it confirm its stoichiometry, but it also clearly highlights the crystallographic inequivalence of the two carboxylic groups of MLOA in the adduct. One group is present as a carboxylate (175.5 ppm), while the other is a carboxylic acid (170.4 ppm) establishing an H-bond with another MLOA molecule. The ^13^C CPMAS spectrum of **SULP-AZA** shows the presence of a new crystalline phase exhibiting in its asymmetric unit one SULP and one AZA molecule. However, since the main supramolecular interactions involve NH/NH_2_ donors and N acceptor groups (see Figure 7), the ^15^N CPMAS spectrum is also particularly informative (see Figure S38 in the Supplementary Materials). It is indeed possible to confirm the formation of the molecular salt since the N4*′* signal of the acid group of AZA shifts by 53 ppm towards higher frequencies, in perfect agreement with the chemical shift values reported in the literature upon the deprotonation of amides bound to strongly electron-attracting groups [39]. In contrast, for the **SULP-MLEA** and **SULP-IND** adducts, where the ^13^C and ^15^N chemical shifts are ambiguous, ab initio calculations proved crucial to discriminate with certainty between salt or cocrystal formation. For instance, in the case of SULP-MLEA the N3 signal undergoes a high-frequency shift of 1 ppm on passing from a N3∙∙∙H_2_N21 to N3^+^-H, while usually a larger shift is expected (≈5 ppm) [40]. The ^15^N CPMAS spectra are reported in the Supplementary Materials (Figure S39).

The results of the DFT optimizations suggested salt formation in all the crystal structures of the SULP adducts since in each of the lowest-energy structures the hydrogen is bonded to N3. All optimized lattice parameters and RMSDs are reported in Table S2 in the Supplementary Materials. Concerning **SULP-MLEA** and **SULP-IND**, the computed chemical shifts confirm the experimental ambiguous positions of the ^13^C and ^15^N signals related to the COO^−^ and N3 groups. This indicates that, in these two particular cases, other effects (e.g., packing, electronics, etc.) concur to the observed unexpected positions. Complete ^13^C and ^15^N experimental and computed chemical shifts and relative RMSEs for all SULP adducts are shown in Tables S4–S9 in the Supplementary Materials.

Furthermore, by comparing the chemical shift values of the carboxylic carbon of the pure coformer with those recorded for **SULP-PABA**, **SULP-CAFA**, **SULP-NA,** and **SULP-SA** adducts (from 174.4 to 176.2 ppm; from 175.5 to 178.9 ppm; from 167.3 to 170.6 ppm; from 180.3 to 181.0 ppm, respectively), the formation of the molecular salts could be deduced, as a result of protonic transfer from the acid to SULP. The stochiometric API:coformer ratio is 1:1 for **SULP-CAFA**, **SULP-NA,** and **SULP-SA**, while **SULP-PABA** is characterized by two independent molecules of PABA and two independent molecules of SULP in the unit cell, as observed from the characteristic splitting of all ^13^C signals. The identification of the protonation state of the **SULP-MLIA** adduct is more challenging: two SULP molecules and two MLIA molecules are clearly present in the asymmetric unit. Due to the shape and intensity of the signal related to the carboxylic group, it is assumed that there is one protonated acid group (177.8 ppm) and three deprotonated acid groups (180.5 ppm). Thus, the adduct could be better defined as a salt cocrystal since it is composed by both salified and neutral molecules in its unit cell. Finally, by evaluating the shift of the carboxylic carbon in the ^13^C CPMAS spectrum of **SULP-IBU**, the formation of a new cocrystal form, with a stoichiometric ratio of 1:1, was detected. In fact, the signal moves toward lower frequencies from 182.9 to 179.2 ppm. The chemical shift reported in the literature for the protonated carboxylic group of IBU, involved in an H-bond interaction with a nitrogen atom, in an (IBU)_2_(4,4*′*-bipyridyl) cocrystal is 177 ppm [41], a value very close to that recorded in our case. Consequently, the establishment of an interaction via H-bonding involving the H-bond donor carboxylic group of IBU and an H-bond acceptor site of SULP, most likely the N3 group, was surmised, resulting in the breakup of the IBU COOH···COOH homosynthon, present in pure IBU, in favor of the formation of a COOH···N heterosynthon.

### 3.3. In Vitro Dissolution Tests

**Figure 9:**
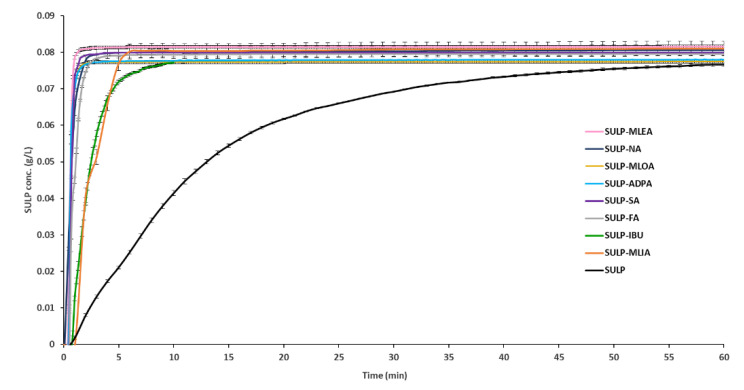
Dissolution profiles of pure SULP (black) and adducts: **SULP-ADPA** (light blue), **SULP-FA** (grey), **SULP-MLEA** (pink), **SULP-MLIA** (orange), **SULP-MLOA** (yellow), **SULP-NA** (blue), **SULP-SA** (purple), **SULP-IBU** (green).

SULP concentration during dissolution tests was quantified by UV absorbance at 291 nm; UV scans from 500 to 190 nm were carried out to check the UV profiles. Dissolution tests were conducted only on **SULP-ADPA**, **SULP-FA**, **SULP-MLEA**, **SULP-MLIA**, **SULP-MLOA**, **SULP-NA**, **SULP-SA,** and **SULP-IBU** since for the others the absorbances of pure coformers, INDO, AZA, CAFA, and PABA, lay on the same wavelength of SULP. Pure SULP dissolves almost completely within 1 h, reaching concentrations of approximately 0.08 g/L, while all the new crystal forms completely dissolve in less than 10 min, as visible from Figure 9. More specifically, **SULP-ADPA**, **SULP-FA**, **SULP-MLEA**, **SULP-MLOA**, **SULP-NA,** and **SULP-SA** exhibit almost superposable dissolution profiles, dissolving the entire drug amount within 3 min, whereas **SULP-IBU** and **SULP-MLIA** are solubilized in 10 min. No recrystallization events are evident over 1 h of analysis, attesting to the stability in the solution. It is noteworthy that the gain in the dissolution rate is neither dependent on the drug-to-coformer stoichiometry nor the water affinity of the coformer. In fact, the highly soluble MLIA provides one of the slowest performances, almost comparable to that of IBU, a poorly soluble API. Furthermore, SA and FA, although present in a lower concentration, offer the same dissolution enhancement as equimolar (or even more concentrated) coformers. The features of the adducts in solution appear more determined by the drug ionic state rather than the adduct components (i.e., coformers). In fact, the cocrystals (e.g., **SULP-IBU**) are slightly slower, while the saline forms (e.g., **SULP-ADPA**) are faster in their dissolution. The proposed SULP dissolution enhancement, achieved by creating multicomponent systems, is particularly promising for developing solid oral dosage forms. It is reasonable to assume that the dissolution improvement also results in a bioavailability enhancement, which ultimately leads to a reduction in the therapeutic dose and the side effects, characteristics that need to be verified with appropriate in vivo studies, which go well beyond the aim of the paper.

## 4. Conclusions

The main focus of this work was the design of new salts and cocrystals of SULP aimed at improving the dissolution rate and water solubility of this API. Experimental screening was then carried out using different promising coformers for the formation of supramolecular synthons with SULP. Twelve new multicomponent crystal forms were obtained: three co-drugs of which two are molecular salts and one is a cocrystal; a salt cocrystal; and eight molecular salts. All samples were characterized in the solid state by complementary techniques to evaluate the ionic or neutral character of the adducts. For **SULP-ADPA**, **SULP-FA**, **SULP-MLEA**, **SULP-MLOA,** and **SULP-IND** it was possible to solve their crystal structures via SCXRD. Among the 12 selected coformers, some are of particular pharmacological interest because they are GRAS molecules with positive biological effects on human health. For example, NA is a water soluble and essential vitamin of the B group, SA has a role as a nutraceutical, anti-ulcer drug, micronutrient, and key metabolite, while CAFA acts as an antioxidant and prevents oxidative stress. In addition, other coformers are currently in use as common pharmaceutical excipients [42] (e.g., ADPA, MLIA, FA, and MLEA); this suggests that the binary cocrystals can constitute a premixed functional product. Although it is unlikely that the adducts with AZA, IBU, and IND will find clinical application, the provided results represent an effective way to use supramolecular synthons for obtaining co-drugs that are generally interesting whenever drug coadministration is relevant for a pathology [43]. This paper shows how it was possible to overcome the drawbacks of SULP through the design of new crystal forms. Indeed, all adducts have remarkable dissolution properties with respect to pure SULP that are very promising for eventual commercial use.

## 5. Patent

The following patent was filed, resulting from the work reported in this manuscript: R. Gobetto, M. R. Chierotti, R. Birolo, F. Bravetti, S. Bordignon, *Crystalline Compounds of Sulpiride* (Nr. 102022000011363).

## Figures and Tables

**Table 1:** Crystal data and structure refinement details for **SULP-ADPA**, **SULP-FA**, **SULP-MLEA**, **SULP-MLOA**, **SULP-IND,** and **SULP-AZA**.

Identification code	SULP-FA	SULP-ADPA	SULP-MLEA	SULP-MLOA	SULP-IND	SULP-AZA
Empirical formula	C_34_H_50_N_6_O_12_S_2_	C_36_H_56_N_6_O_12_S_2_	C_19_H_27_N_3_O_8_S	C_18_H_27_N_3_O_8_S	C_34_H_41_ClN_4_O_9_S	C_19_H_29_N_7_O_7_S_3_
Formula weight	798.92	831.00	457.49	445.48	717.22	563.67
Temperature/K	200.00	150.00	200.00	200.00	200.00	200.00
Crystal system	triclinic	triclinic	monoclinic	monoclinic	triclinic	monoclinic
Space group	$P\bar{1}$	$P\bar{1}$	*Pn*	*P*2_1_/*c*	$P\bar{1}$	*P*2_1_/*c*
*a*/Å	7.3227(9)	7.2641(4)	7.9084(5)	7.4255(2)	7.2881(13)	9.6257(4)
*b*/Å	8.0884(10)	8.2222(4)	10.8794(7)	35.7096(11)	8.0909(17)	11.5202(5)
*c*/Å	16.641(2)	17.7641(9)	12.4918(8)	8.1883(3)	29.704(6)	22.5198(10)
α/°	81.355(4)	84.511(3)	90	90	93.651(6)	90
β/°	86.017(4)	82.479(3)	92.660(2)	102.8120(10)	92.351(5)	98.676(3)
γ/°	76.489(4)	78.245(3)	90	90	101.309(5)	90
Volume/Å^3^	946.9(2)	1027.20(9)	1073.62(12)	2117.17(12)	1711.5(6)	2468.65(19)
Z	1	1	2	4	2	4
ρ_calc_g/cm^3^	1.401	1.343	1.415	1.398	1.392	1.517
μ/mm^−1^	1.871	1.743	0.202	1.806	0.233	3.237
F(000)	424.0	444.0	484.0	944.0	756.0	1184.0
Crystal size/mm^3^	0.04 × 0.03 × 0.03	0.04 × 0.03 × 0.01	0.07 × 0.02 × 0.02	0.05 × 0.05 × 0.04	0.07 × 0.06 × 0.04	0.05 × 0.04 × 0.02
Radiation	CuKα (λ = 1.54178)	CuKα (λ = 1.54178)	MoKα (λ = 0.71073)	CuKα (λ = 1.54178)	MoKα (λ = 0.71073)	CuKα (λ = 1.54178)
2Θ range for data collection/°	5.374 to 89.052	5.03 to 149.294	4.968 to 52.826	4.95 to 150.01	4.128 to 52.998	7.942 to 150.688
Index ranges	−6 ≤ h ≤ 6 −7 ≤ k ≤ 7 −15 ≤ l ≤ 15	−8 ≤ h ≤ 9 −10 ≤ k ≤ 10 −22 ≤ l ≤ 22	−9 ≤ h ≤ 9 −13 ≤ k ≤ 13−15 ≤ l ≤ 15	−9 ≤ h ≤ 8 −44 ≤ k ≤ 44 −10 ≤ l ≤ 10	−9 ≤ h ≤ 8 −10 ≤ k ≤ 10 −37 ≤ l ≤ 37	−11 ≤ h ≤ 11 0 ≤ k ≤ 14 0 ≤ l ≤ 28
Reflections collected	3913	18659	19091	44139	49909	5017
Independent reflections	1443 R_int_ = 0.0701, R_sigma_ = 0.1034	4182 R_int_ = 0.0601, R_sigma_ = 0.0426	4278 R_int_ = 0.0830, R_sigma_ = 0.0684	4351 R_int_ = 0.0422, R_sigma_ = 0.0219	7033 R_int_ = 0.1496, R_sigma_ = 0.0970	5017 R_int_ = n/a, R_sigma_ = 0.0700
Data/restraints/parameters	1443/0/217	4182/0/257	4278/2/298	4351/0/282	7033/0/449	5017/0/333
Goodness-of-fit on F^2^	1.017	1.021	1.071	1.167	1.057	1.075
Final R indexes [I>=2σ (I)]	R_1_ = 0.0762, wR_2_ = 0.1885	R_1_ = 0.0460, wR_2_ = 0.1174	R_1_ = 0.0510, wR_2_ = 0.0985	R_1_ = 0.0808, wR_2_ = 0.1926	R_1_ = 0.1235, wR_2_ = 0.3402	R_1_ = 0.1036, wR_2_ = 0.2760
Final R indexes [all data]	R_1_ = 0.1462, wR_2_ = 0.2413	R_1_ = 0.0618, wR_2_ = 0.1282	R_1_ = 0.0731, wR_2_ = 0.1067	R_1_ = 0.0822, wR_2_ = 0.1933	R_1_ = 0.1846, wR_2_ = 0.3812	R_1_ = 0.1356, wR_2_ = 0.3064
Largest diff. peak/hole / e Å^−3^	0.86/−0.34	0.41/−0.69	0.21/−0.23	0.84/−0.59	1.19/−0.55	0.94/−0.62

## Data Availability

Not applicable.

## Supplementary Materials

The following are available online at https://www.mdpi.com/article/10.3390/pharmaceutics14091754/s1. Table S1. Synthetic conditions for the preparation of the adducts; Table S2. Lattice parameters and RMSD20 of the optimised crystal structures; Figures S1–S12. FTIR-ATR spectra; Figures S13–S24. Experimental X-ray powder patterns; Figures S25–S30. Experimental and calculated X-ray powder diffractograms; Figures S31–S36. ORTEP diagrams; Table S3. H-bonds interaction for all the crystal structures; Figure S37. ^13^C CPMAS spectra of the 12 coformers; Figures S38 and S39. ^15^N CPMAS spectra of the adducts; Tables S4–S9 ^13^C and ^15^N experimental and computed chemical shifts and relative RMSEs for the adducts.

## References

[1] Eisenegger C., Naef M., Linssen A., Clark L., Gandamaneni P.K., Müller U., Robbins T.W. (2014). Role of Dopamine D2 Receptors in Human Reinforcement Learning. Neuropsychopharmacology.

[2] Lai E.C.-C., Chang C.-H., Kao Yang Y.-H., Lin S.-J., Lin C.-Y. (2013). Effectiveness of Sulpiride in Adult Patients With Schizophrenia. Schizophr. Bull..

[3] Tsume Y., Mudie D.M., Langguth P., Amidon G.E., Amidon G.L. (2014). The Biopharmaceutics Classification System: Subclasses for in Vivo Predictive Dissolution (IPD) Methodology and IVIVC. Eur. J. Pharm. Sci. Off. J. Eur. Fed. Pharm. Sci..

[4] Watanabe K., Sawano T., Terada K., Endo T., Sakata M., Sato J. (2002). Studies on Intestinal Absorption of Sulpiride. (1): Carrier-Mediated Uptake of Sulpiride in the Human Intestinal Cell Line Caco-2. Biol. Pharm. Bull..

[5] Ayoub A.M., Ibrahim M.M., Abdallah M.H., Mahdy M.A. (2016). Sulpiride Microemulsions as Antipsychotic Nasal Drug Delivery Systems: In-Vitro and Pharmacodynamic Study. J. Drug Deliv. Sci. Technol..

[6] M’bitsi-Ibouily G.C., Marimuthu T., du Toit L.C., Kumar P., Choonara Y.E. (2021). In Vitro, Ex Vivo and in Vivo Evaluation of a Novel Metal-Liganded Nanocomposite for the Controlled Release and Improved Oral Bioavailability of Sulpiride. J. Drug Deliv. Sci. Technol..

[7] Mohyeldin S.M., Samy W.M., Ragab D., Abdelmonsif D.A., Aly R.G., Elgindy N.A. (2021). Precisely Fabricated Sulpiride-Loaded Nanolipospheres with Ameliorated Oral Bioavailability and Antidepressant Activity. Int. J. Nanomed..

[8] Tawfeek H.M., Hassan Y.A., Aldawsari M.F., Fayed M.H. (2020). Enhancing the Low Oral Bioavailability of Sulpiride via Fast Orally Disintegrating Tablets: Formulation, Optimization and In Vivo Characterization. Pharmaceuticals.

[9] Grothe E., Meekes H., Vlieg E., ter Horst J.H., de Gelder R. (2016). Solvates, Salts, and Cocrystals: A Proposal for a Feasible Classification System. Cryst. Growth Des..

[10] Aitipamula S., Banerjee R., Bansal A.K., Biradha K., Cheney M.L., Choudhury A.R., Desiraju G.R., Dikundwar A.G., Dubey R., Duggirala N. (2012). Polymorphs, Salts, and Cocrystals: What’s in a Name?. Cryst. Growth Des..

[11] Reddy D.S., Craig D.C., Desiraju G.R. (1996). Supramolecular Synthons in Crystal Engineering. 4. Structure Simplification and Synthon Interchangeability in Some Organic Diamondoid Solids. J. Am. Chem. Soc..

[12] Cruz-Cabeza A.J. (2012). Acid–Base Crystalline Complexes and the PKa Rule. CrystEngComm.

[13] Aramini A., Bianchini G., Lillini S., Bordignon S., Tomassetti M., Novelli R., Mattioli S., Lvova L., Paolesse R., Chierotti M.R. (2021). Unexpected Salt/Cocrystal Polymorphism of the Ketoprofen–Lysine System: Discovery of a New Ketoprofen–l-Lysine Salt Polymorph with Different Physicochemical and Pharmacokinetic Properties. Pharmaceuticals.

[14] Bernasconi D., Bordignon S., Rossi F., Priola E., Nervi C., Gobetto R., Voinovich D., Hasa D., Duong N.T., Nishiyama Y. (2020). Selective Synthesis of a Salt and a Cocrystal of the Ethionamide–Salicylic Acid System. Cryst. Growth Des..

[15] Dai X.-L., Chen J.-M., Lu T.-B. (2018). Pharmaceutical Cocrystallization: An Effective Approach to Modulate the Physicochemical Properties of Solid-State Drugs. CrystEngComm.

[16] Almarsson Ö., Peterson M.L., Zaworotko M. (2012). The A to Z of Pharmaceutical Cocrystals: A Decade of Fast-Moving New Science and Patents. Pharm. Pat. Anal..

[17] Kavanagh O.N., Croker D.M., Walker G.M., Zaworotko M.J. (2019). Pharmaceutical Cocrystals: From Serendipity to Design to Application. Drug Discov. Today.

[18] Raheem Thayyil A., Juturu T., Nayak S., Kamath S. (2020). Pharmaceutical Co-Crystallization: Regulatory Aspects, Design, Characterization, and Applications. Adv. Pharm. Bull..

[19] Deka P., Gogoi D., Althubeiti K., Rao D.R., Thakuria R. (2021). Mechanosynthesis, Characterization, and Physicochemical Property Investigation of a Favipiravir Cocrystal with Theophylline and GRAS Coformers. Cryst. Growth Des..

[20] Sinthupoom N., Prachayasittikul V., Prachayasittikul S., Ruchirawat S., Prachayasittikul V. (2015). Nicotinic Acid and Derivatives as Multifunctional Pharmacophores for Medical Applications. Eur. Food Res. Technol..

[21] Jung U.J., Lee M.-K., Park Y.B., Jeon S.-M., Choi M.-S. (2006). Antihyperglycemic and Antioxidant Properties of Caffeic Acid in Db/Db Mice. J. Pharmacol. Exp. Ther..

[22] Rodrigues M., Lopes J., Sarraguça M. (2018). Vibrational Spectroscopy for Cocrystals Screening. A Comparative Study. Molecules.

[23] Haskins M.M., Zaworotko M.J. (2021). Screening and Preparation of Cocrystals: A Comparative Study of Mechanochemistry vs Slurry Methods. Cryst. Growth Des..

[24] Sheldrick G.M. (2015). SHELXT-Integrated Space-Group and Crystal-Structure Determination. Acta Cryst A.

[25] Sheldrick G.M. (2015). Crystal Structure Refinement with SHELXL. Acta Cryst Sec C.

[26] Dolomanov O.V., Bourhis L.J., Gildea R.J., Howard J.A.K., Puschmann H. (2009). OLEX2: A Complete Structure Solution, Refinement and Analysis Program. J. Appl. Crystallogr..

[27] Kohri N., Naasani I., Iseki K., Miyazaki K. (2011). Improving the Oral Bioavailability of Sulpiride by a Gastric-Retained Form in Rabbits. J. Pharm. Pharmacol..

[28] Giannozzi P., Baroni S., Bonini N., Calandra M., Car R., Cavazzoni C., Ceresoli D., Chiarotti G.L., Cococcioni M., Dabo I. (2009). QUANTUM ESPRESSO: A Modular and Open-Source Software Project for Quantum Simulations of Materials. J. Phys. Condens. Matter.

[29] Lee K., Murray É.D., Kong L., Lundqvist B.I., Langreth D.C. (2010). Higher-Accuracy van der Waals Density Functional. Phys. Rev. B.

[30] Perdew J.P., Yue W. (1986). Accurate and Simple Density Functional for the Electronic Exchange Energy: Generalized Gradient Approximation. Phys. Rev. B.

[31] Perdew J.P., Burke K., Ernzerhof M. (1996). Generalized Gradient Approximation Made Simple. Phys. Rev. Lett..

[32] Franco F., Baricco M., Chierotti M.R., Gobetto R., Nervi C. (2013). Coupling Solid-State NMR with GIPAW Ab Initio Calculations in Metal Hydrides and Borohydrides. J. Phys. Chem. C.

[33] Pickard C.J., Mauri F. (2001). All-Electron Magnetic Response with Pseudopotentials: NMR Chemical Shifts. Phys. Rev. B.

[34] Reddy G.N.M., Huqi A., Iuga D., Sakurai S., Marsh A., Davis J.T., Masiero S., Brown S.P. (2017). Co-Existence of Distinct Supramolecular Assemblies in Solution and in the Solid State. Chem.-Eur. J..

[35] Macrae C.F., Sovago I., Cottrell S.J., Galek P.T.A., McCabe P., Pidcock E., Platings M., Shields G.P., Stevens J.S., Towler M. (2020). Mercury 4.0: From Visualization to Analysis, Design and Prediction. J. Appl. Crystallogr..

[36] Rossi F., Cerreia Vioglio P., Bordignon S., Giorgio V., Nervi C., Priola E., Gobetto R., Yazawa K., Chierotti M.R. (2018). Unraveling the Hydrogen Bond Network in a Theophylline–Pyridoxine Salt Cocrystal by a Combined X-Ray Diffraction, Solid-State NMR, and Computational Approach. Cryst. Growth Des..

[37] Cerreia Vioglio P., Chierotti M.R., Gobetto R. (2017). Pharmaceutical Aspects of Salt and Cocrystal Forms of APIs and Characterization Challenges. Adv. Drug Deliv. Rev..

[38] Bordignon S., Cerreia Vioglio P., Amadio E., Rossi F., Priola E., Voinovich D., Gobetto R., Chierotti M.R. (2020). Molecular Crystal Forms of Antitubercular Ethionamide with Dicarboxylic Acids: Solid-State Properties and a Combined Structural and Spectroscopic Study. Pharmaceutics.

[39] Bagno A., Comuzzi C. (1999). Deprotonation of Amides and Polyfunctional Imides Probed by Heteronuclear NMR and Quantum Chemical Calculations. Eur. J. Org. Chem..

[40] Gobetto R., Nervi C., Valfrè E., Chierotti M.R., Braga D., Maini L., Grepioni F., Harris R.K., Ghi P.Y. (2005). 1 H MAS, 15 N CPMAS, and DFT Investigation of Hydrogen-Bonded Supramolecular Adducts between the Diamine 1,4-Diazabicyclo-[2.2.2]Octane and Dicarboxylic Acids of Variable Chain Length. Chem. Mater..

[41] Chen S., Xi H., Henry R.F., Marsden I., Zhang G.G.Z. (2010). Chiral Co-Crystal Solid Solution: Structures, Melting Point Phase Diagram, and Chiral Enrichment of (Ibuprofen)_2_(4,4′-Dipyridyl). CrystEngComm.

[42] Pharmaceutical Press-Handbook of Pharmaceutical Excipients Ninth Edition. https://www.pharmpress.com/product/9780857113757/excipients.

[43] Bordignon S., Cerreia Vioglio P., Priola E., Voinovich D., Gobetto R., Nishiyama Y., Chierotti M.R. (2017). Engineering Codrug Solid Forms: Mechanochemical Synthesis of an Indomethacin–Caffeine System. Cryst. Growth Des..

