# Supplementary material for: Overcoming the Drawbacks of Sulpiride by Means of New Crystal Forms
Source: Pharmaceutics. 2022 Aug 23;14(9):1754. doi: 10.3390/pharmaceutics14091754 (PMC9501926; doi:10.3390/pharmaceutics14091754)
Supplement: Supplementary file 1 [file pharmaceutics-14-01754-s001.zip › pharmaceutics-1841057-supplementary.pdf]

Supplementary Information

Overcoming the Drawbacks of Sulpiride by means of New Crystal Forms

R. Birolo[1], F. Bravetti[1], S. Bordignon[1], I. D'Abbrunzo[2], P. P. Mazzeo[3], B. Perissutti[2], A. Bacchi[3], M. R. Chierotti[1], R. Gobetto[1]

[1]Department of Chemistry, University of Torino, Via P. Giuria 7, 10125 Torino, Italy
[2]Department of Chemical and Pharmaceutical Sciences, University of Trieste, P.le Europa 1, 34127 Trieste, Italy
[3]Department of Chemical, Life and Environmental Sustainability Sciences, University of Parma, Viale delle Scienze 17A, 43124 Parma, Italy

Table of contents	Page
**Table S1.** Synthetic conditions for the preparation of the adducts.	1
**Table S2.** Lattice parameters and $RMSD_{20}$ of the optimised crystal structures.	2
**Figures S1-S12.** FTIR-ATR spectra.	2-6
**Figures S13-S24**. Experimental X-ray powder patterns.	7-12
**Figures S25-S30.** Experimental and calculated X-ray powder diffractograms.	13-15
**Figures S31-S36.** ORTEP diagrams.	16-18
**Table S3.** H-bonds interaction for all the crystal structures.	19
**Figure S37.** ^{13}C CPMAS spectra of the 12 coformers.	19
**Figures S38-S39.** ^{15}N CPMAS spectra of the adducts.	20
**Tables S4-S9** ^{13}C and ^{15}N experimental and computed chemical shifts and relative RMSEs for the adducts.	21-26

Table S1. Synthetic conditions for the preparation of the adducts.

Name	API	Coformer	API:cof	Synthetic Strategy	Solvent
**SULP-ADPA**	SULP	adipic acid	2:1	slurry	ethanol
**SULP-PABA**	SULP	4-aminobenzoic acid	1:1	slurry	acetonitrile
**SULP-CAFA**	SULP	caffeic acid	1:1	slurry	acetonitrile
**SULP-FA**	SULP	fumaric acid	2:1	slurry	ethanol
**SULP-MLEA**	SULP	maleic acid	1:1	slurry	acetonitrile
**SULP-MLIA**	SULP	malic acid	1:1	slurry	ethanol
**SULP-MLOA**	SULP	malonic acid	1:1	slurry	ethanol
**SULP-NA**	SULP	nicotinic acid	1:1	slurry	ethanol
**SULP-SA**	SULP	succinic acid	2:1	slurry	acetonitrile
**SULP-AZA**	SULP	acetazolamide	1:1	slurry	ethanol
**SULP-IBU**	SULP	Ibuprofen	1:1	slurry	methanol
**SULP-IND**	SULP	indomethacin	1:1	slurry	ethanol

Table S2. Lattice parameters and $RMSD_{20}$ of the optimised crystal structures of **SULP-ADPA**, **SULP-FA**, **SULP-MLEA**, **SULP-MLOA**, **SULP-IND** and **SULP-AZA**.

	SULP-ADPA	SULP-FA	SULP-MLEA	SULP-MLOA	SULP-IND	SULP-AZA
**Form**	salt	salt	salt	salt	salt	salt
**a /Å**	7.0710	7.1110	7.7722	7.1891	7.2703	9.6115
**b /Å**	8.1910	8.0557	10.8656	35.4391	7.9850	11.3523
**c /Å**	17.6041	16.4868	12.3374	8.1768	29.0684	22.3570
**α /°**	85.0323	81.6188	90.0000	90.0000	93.7353	90.0000
**β /°**	82.3725	86.4063	94.3452	103.1188	91.5454	99.0724
**γ /°**	78.5038	76.3458	90.0000	90.0000	102.6176	90.0000
**Volume /Å^3**	988.408	907.546	1038.900	2028.880	1641.790	2408.910
**$RMSD_{20}$**	0.144	0.142	0.138	0.160	0.189	0.106

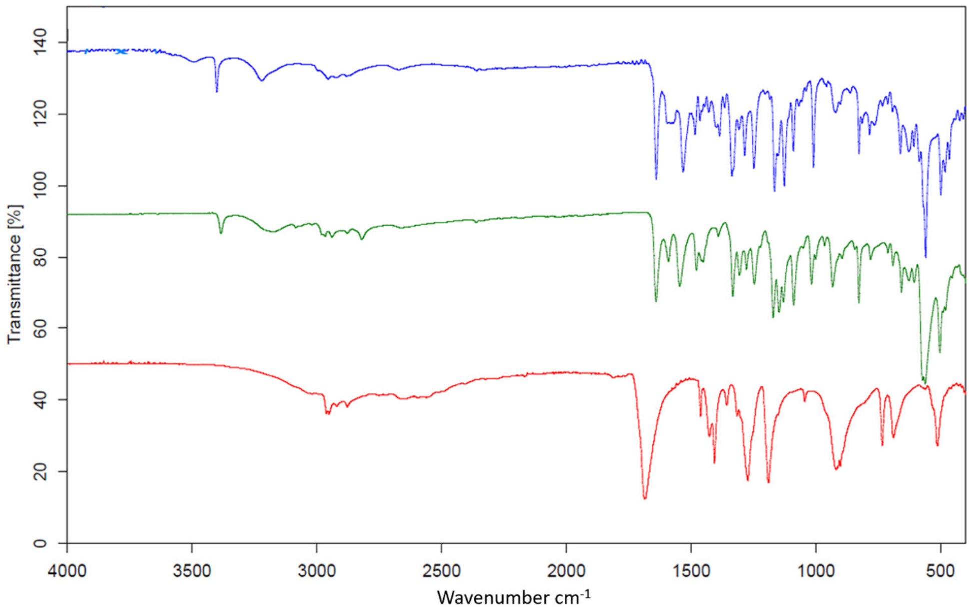

Figure S1. FTIR-ATR spectra of **SUL-ADPA** (blue), SULP (green) and ADPA (red).

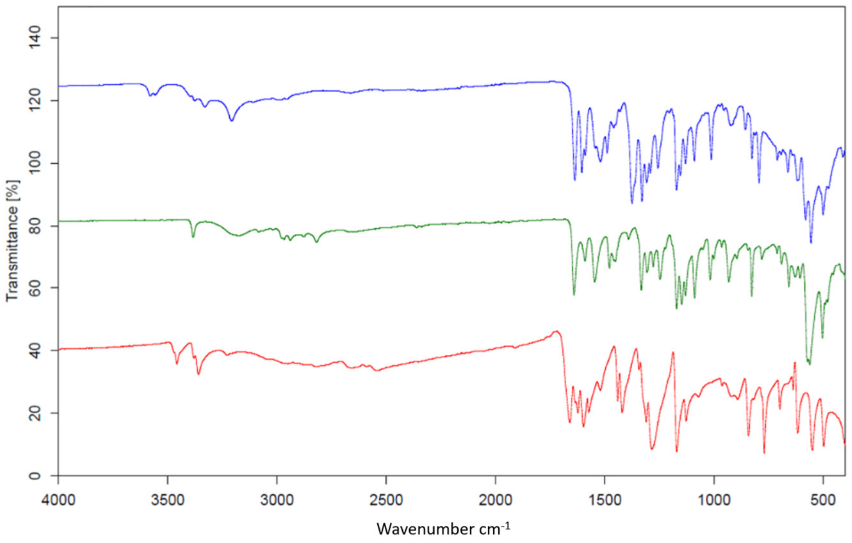

Figure S2. FTIR-ATR spectra of **SUL-PABA** (blue), SULP (green) and PABA (red).

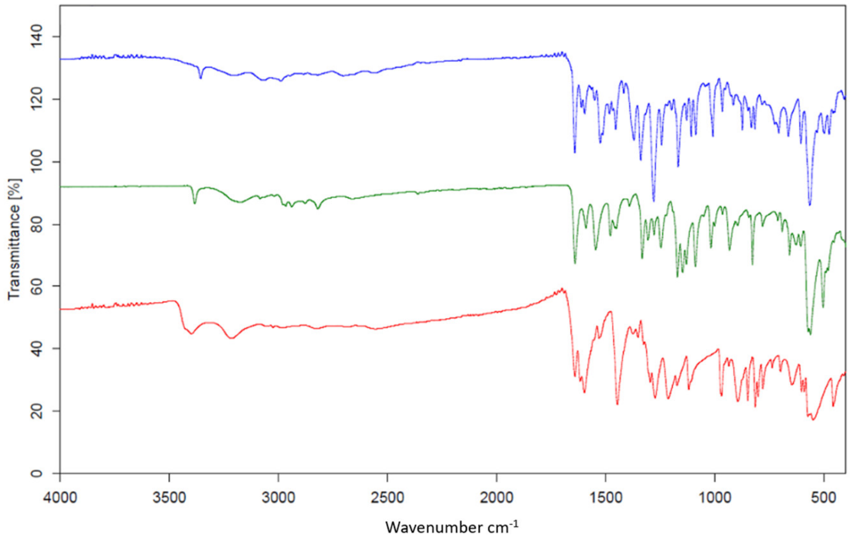

Figure S3. FTIR-ATR spectra of **SUL-CAFA** (blue), SULP (green) and CAFA (red).

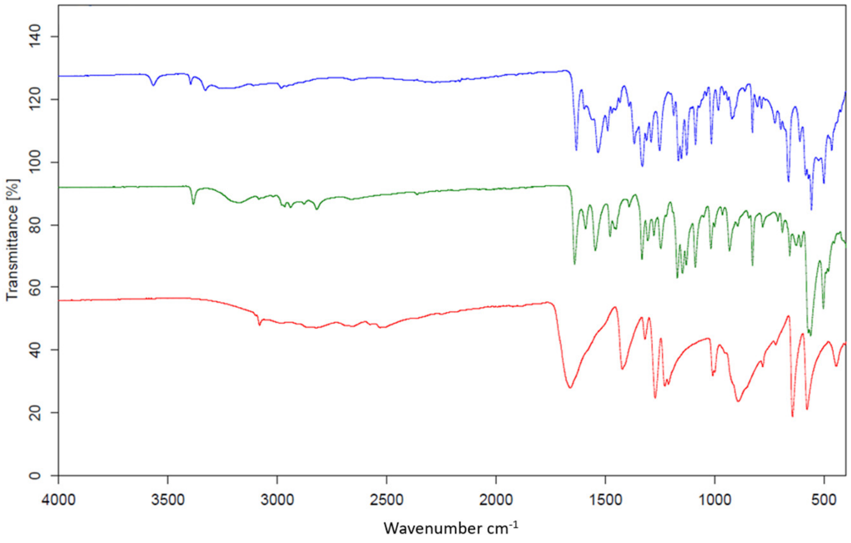

Figure S4. FTIR-ATR spectra of **SUL-FA** (blue), SULP (green) and FA (red).

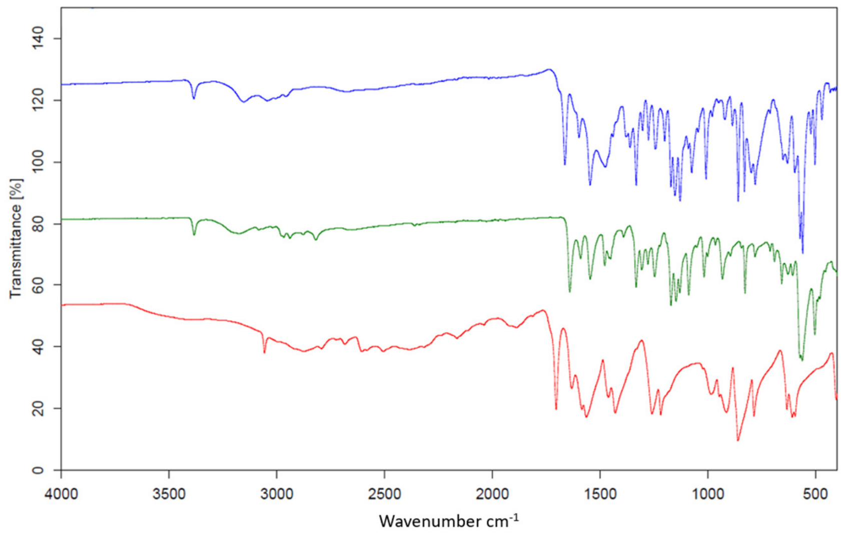

Figure S5. FTIR-ATR spectra of **SUL-MLEA** (blue), SULP (green) and MLEA (red).

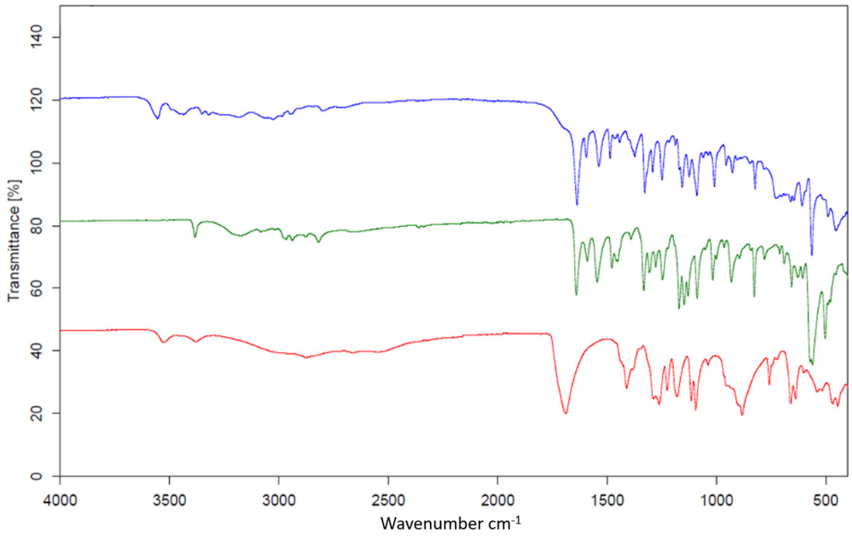

Figure S6. FTIR-ATR spectra of **SUL-MLIA** (blue), SULP (green) and MLIA (red).

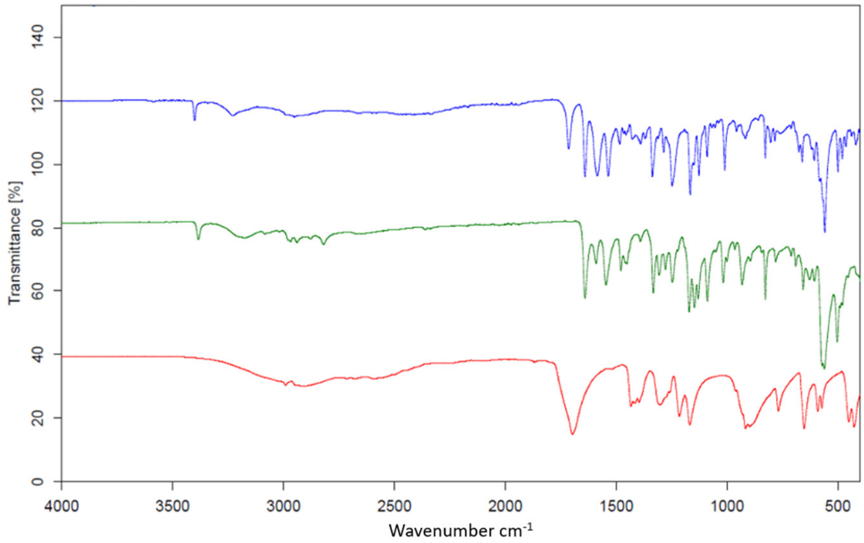

Figure S7. FTIR-ATR spectra of **SUL-MLOA** (blue), SULP (green) and MLOA (red).

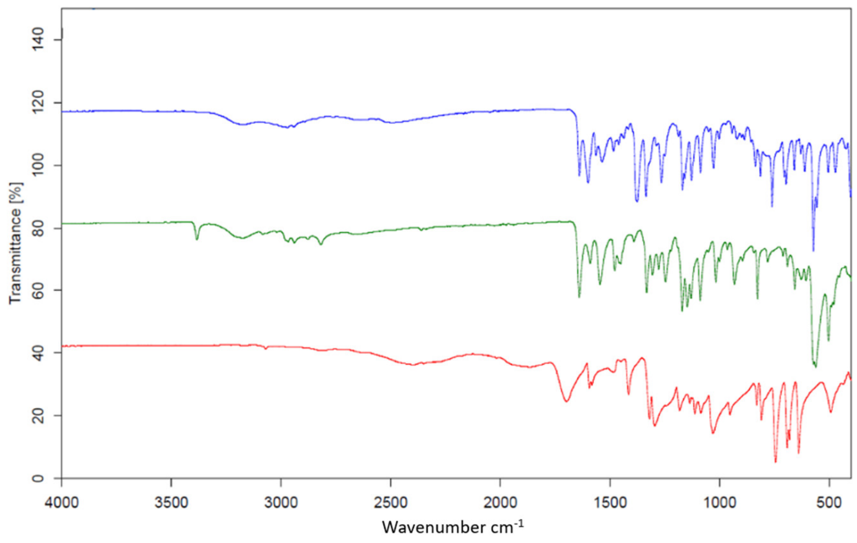

Figure S8. FTIR-ATR spectra of **SUL-NA** (blue), SULP (green) and NA (red).

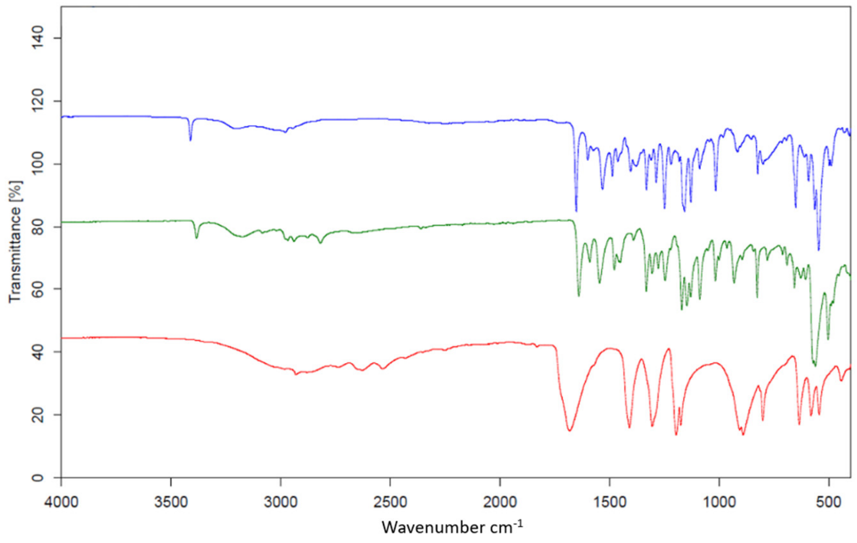

Figure S9. FTIR-ATR spectra of **SUL-SA** (blue), SULP (green) and SA (red).

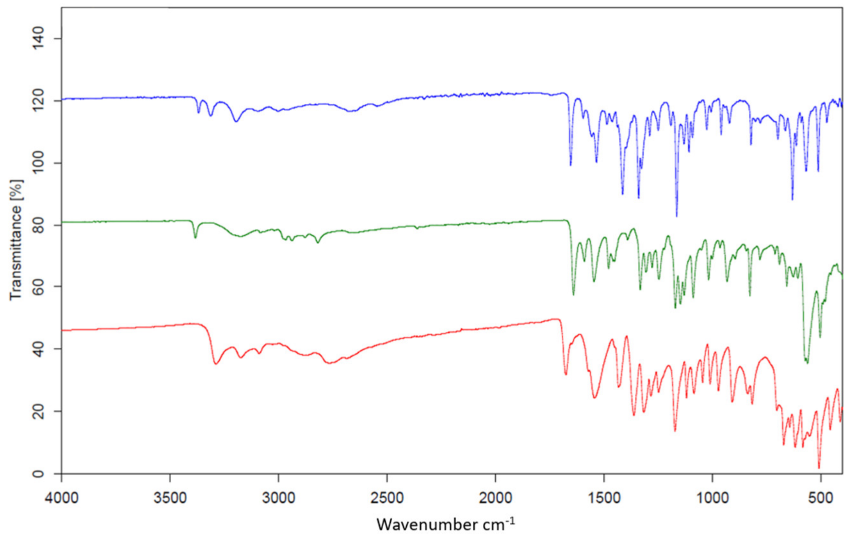

Figure S10. FTIR-ATR spectra of **SUL-AZA** (blue), SULP (green) and AZA (red).

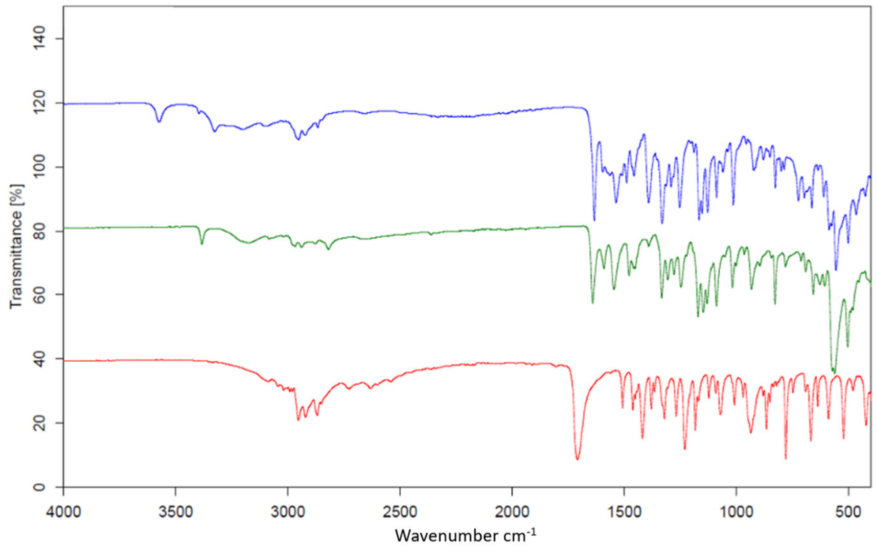

Figure S11. FTIR-ATR spectra of **SUL-IBU** (blue), SULP (green) and IBU (red).

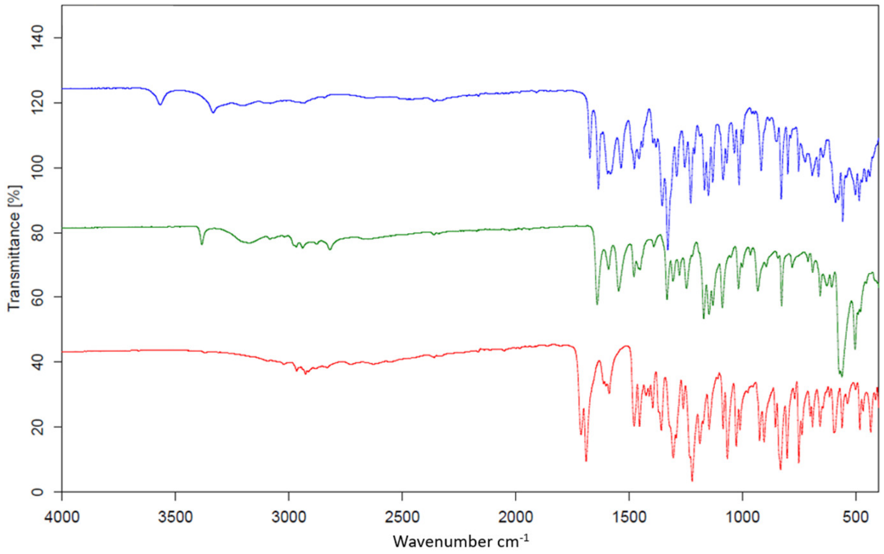

Figure S12. FTIR-ATR spectra of **SUL-IND** (blue), SULP (green) and IND (red).

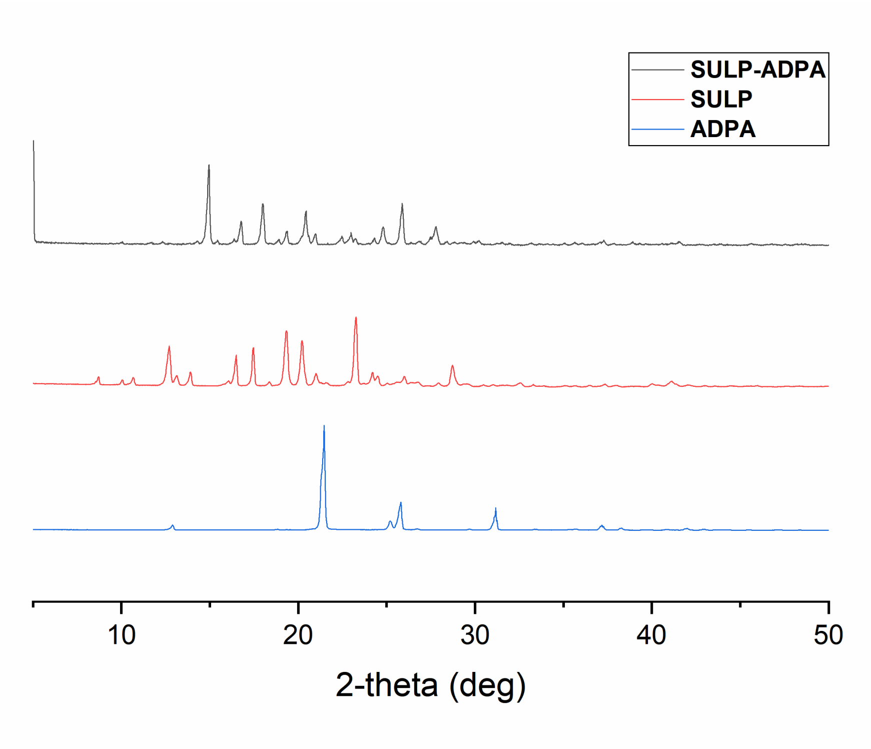

Figure S13. Experimental X-ray powder patterns of **SULP-ADPA** (black), SULP (red), ADPA (blue).

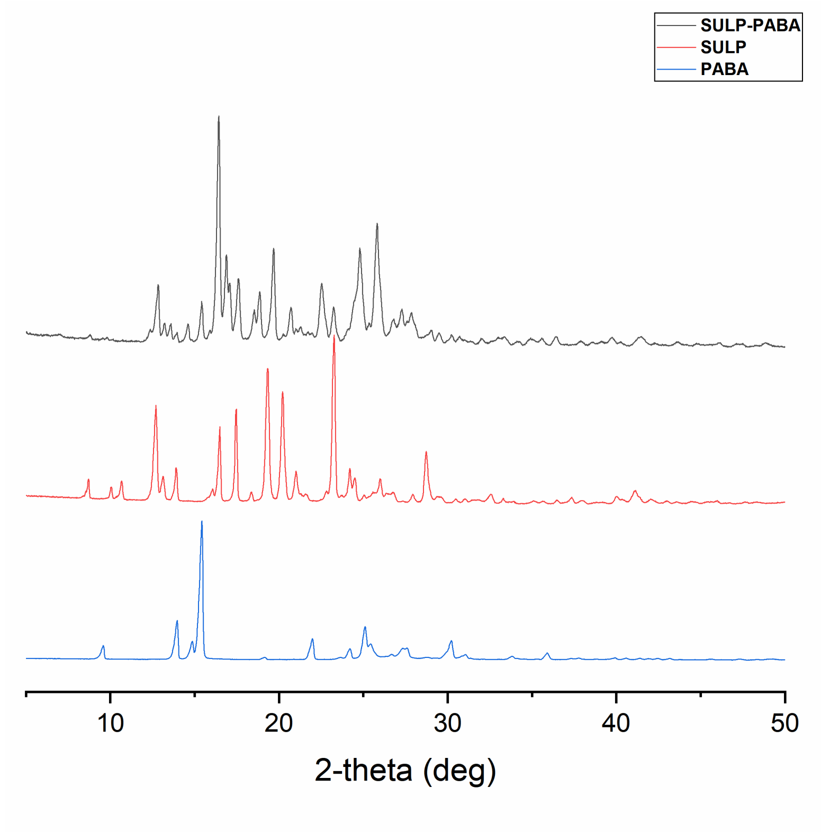

Figure S14. Experimental X-ray powder patterns of **SULP-PABA** (black), SULP (red), PABA (blue).

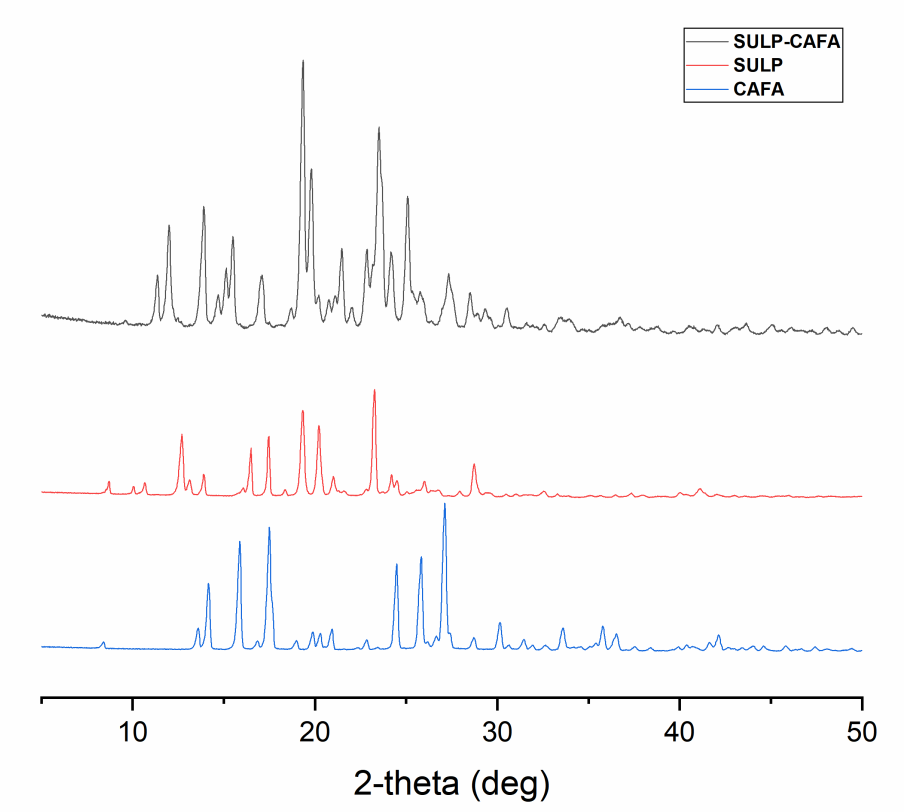

Figure S15. Experimental X-ray powder patterns of **SULP-CAFA** (black), SULP (red), CAFA (blue).

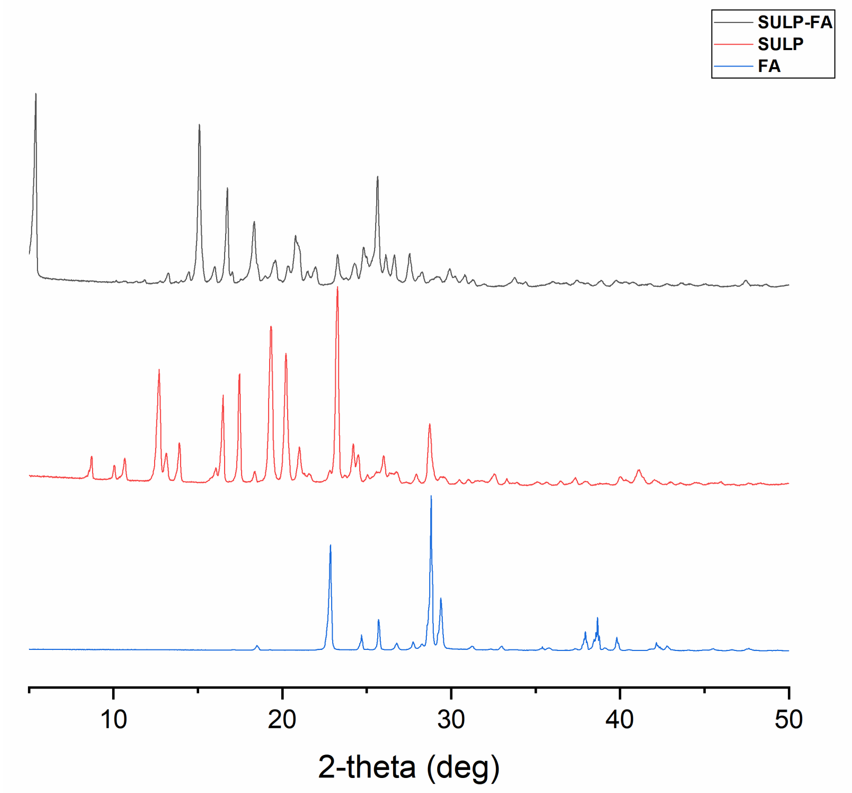

Figure S16. Experimental X-ray powder patterns of **SULP-FA** (black), SULP (red), FA (blue).

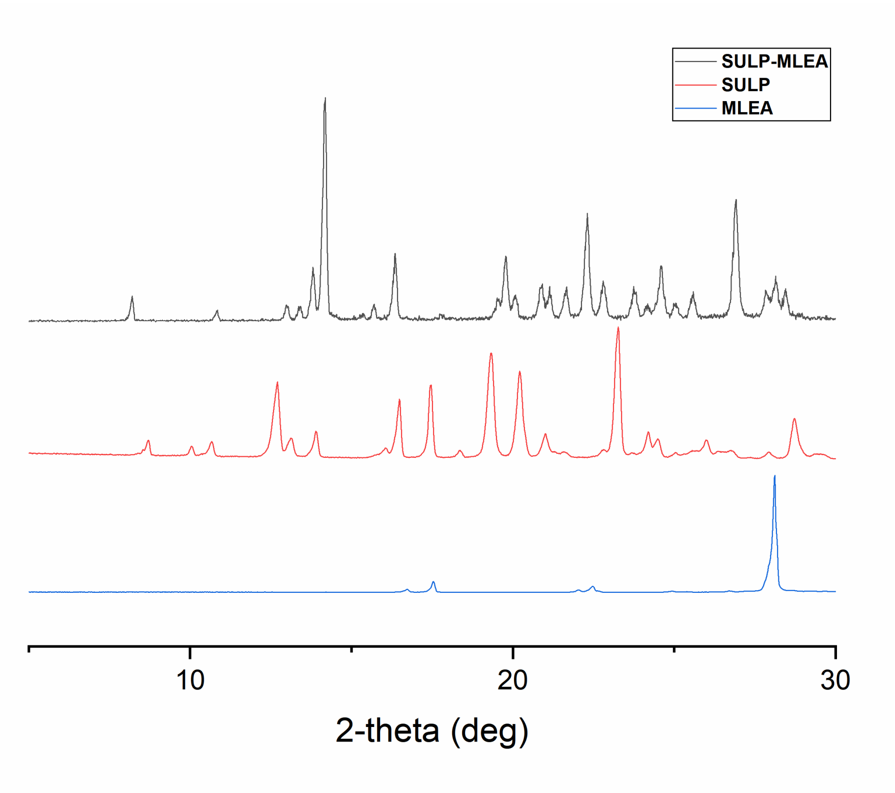

Figure S17. Experimental X-ray powder patterns of **SULP-MLEA** (black), SULP (red), MLEA (blue).

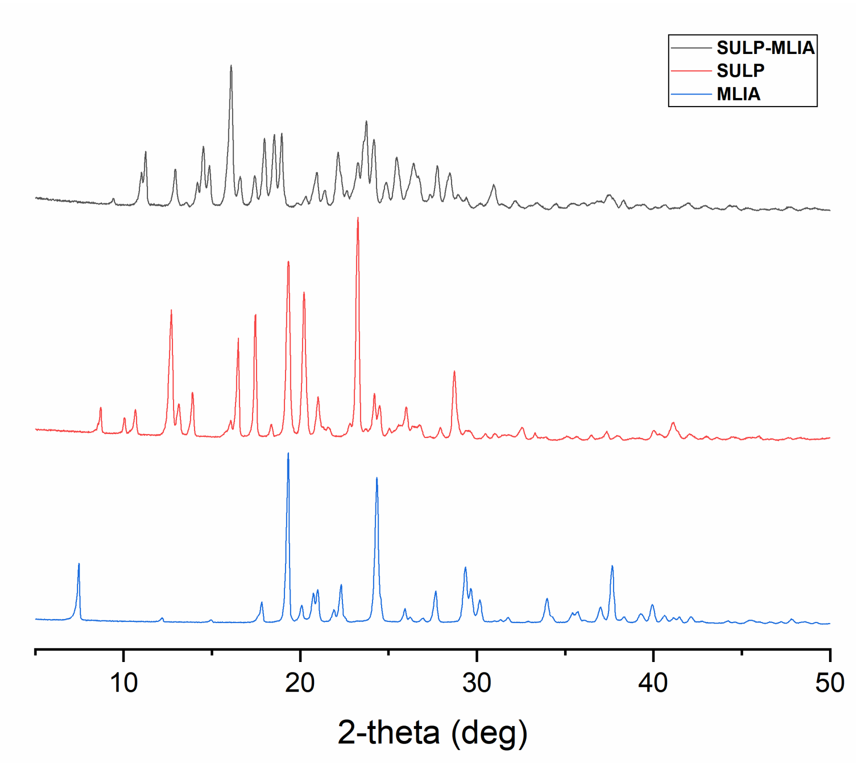

Figure S18. Experimental X-ray powder patterns of **SULP-MLIA** (black), SULP (red), MLIA (blue).

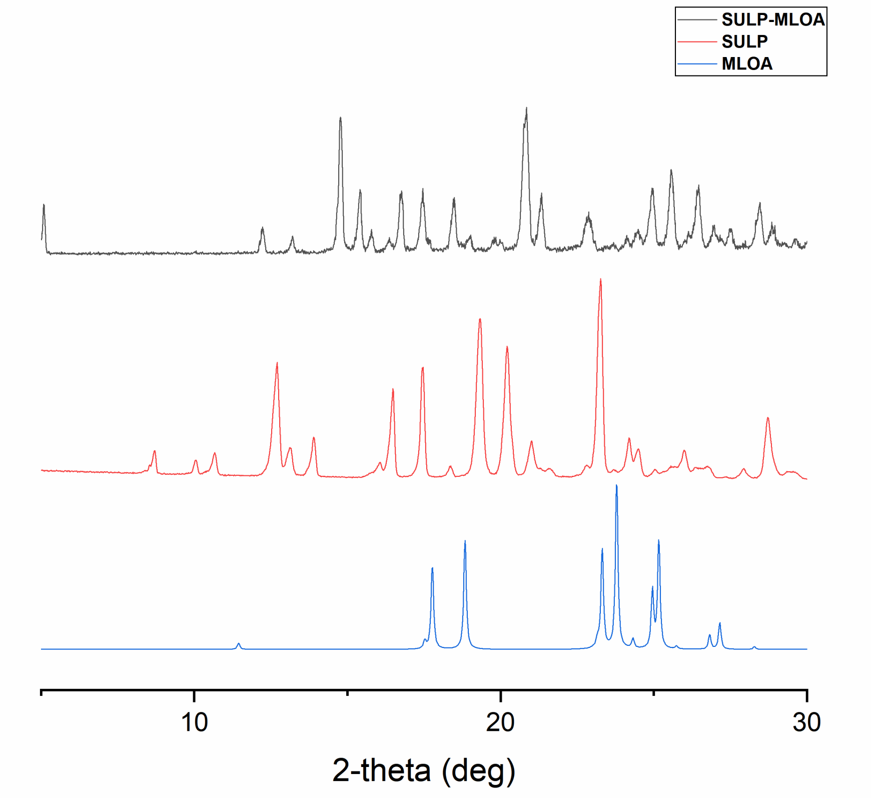

Figure S19. Experimental X-ray powder patterns of **SULP-MLOA** (black), SULP (red), MLOA (blue).

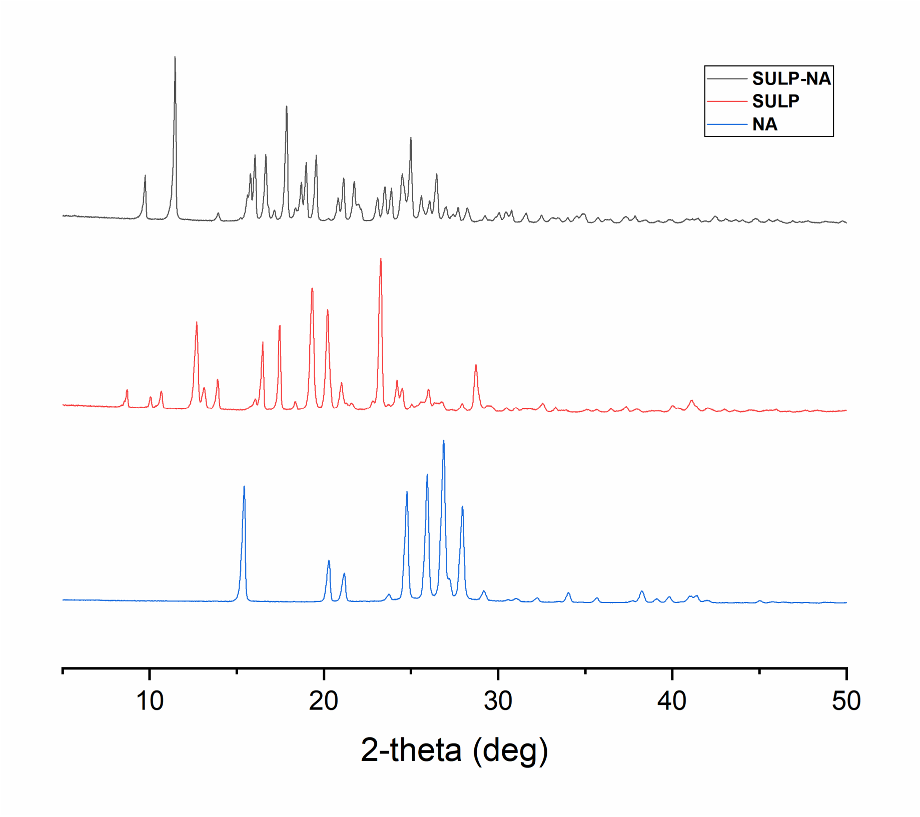

Figure S20. Experimental X-ray powder patterns of **SULP-NA** (black), SULP (red), NA (blue)

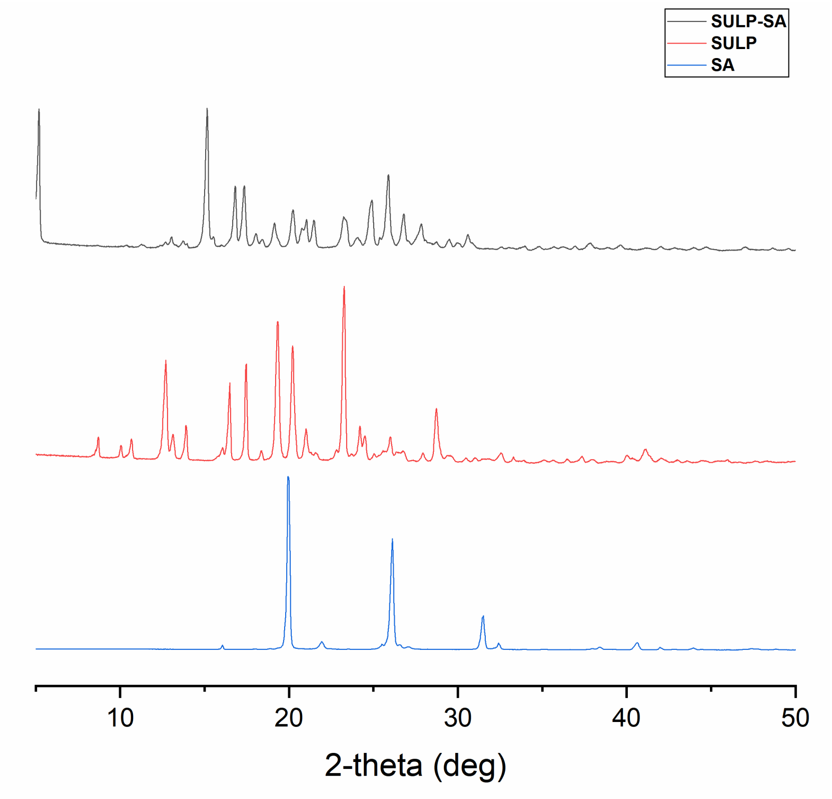

Figure S21. Experimental X-ray powder patterns of **SULP-SA** (black), SULP (red), SA (blue).

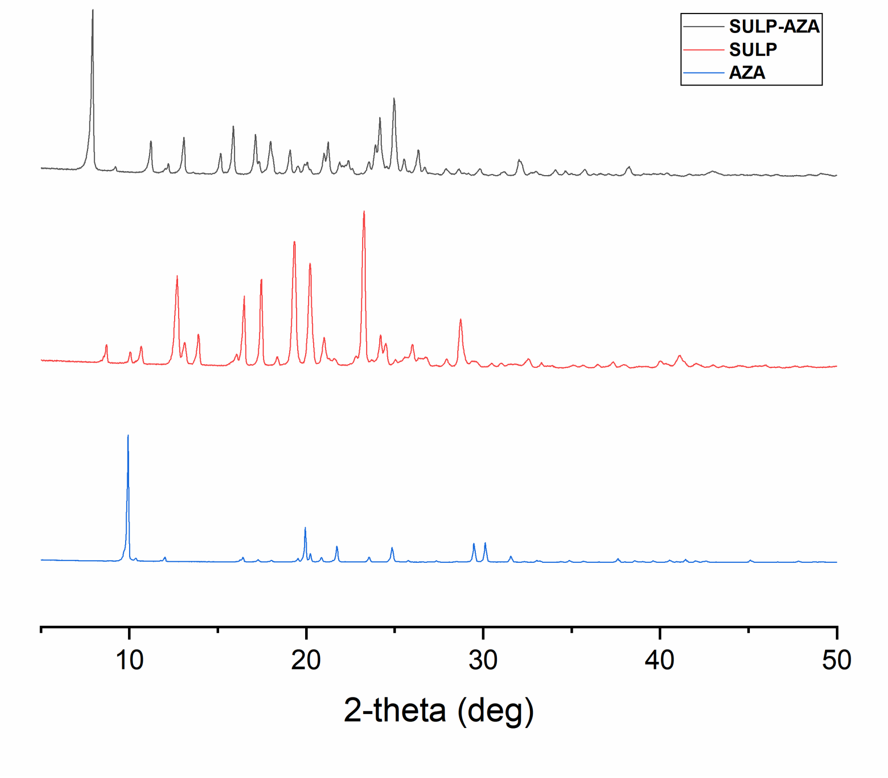

Figure S22. Experimental X-ray powder patterns of **SULP-AZA** (black), SULP (red), AZA (blue).

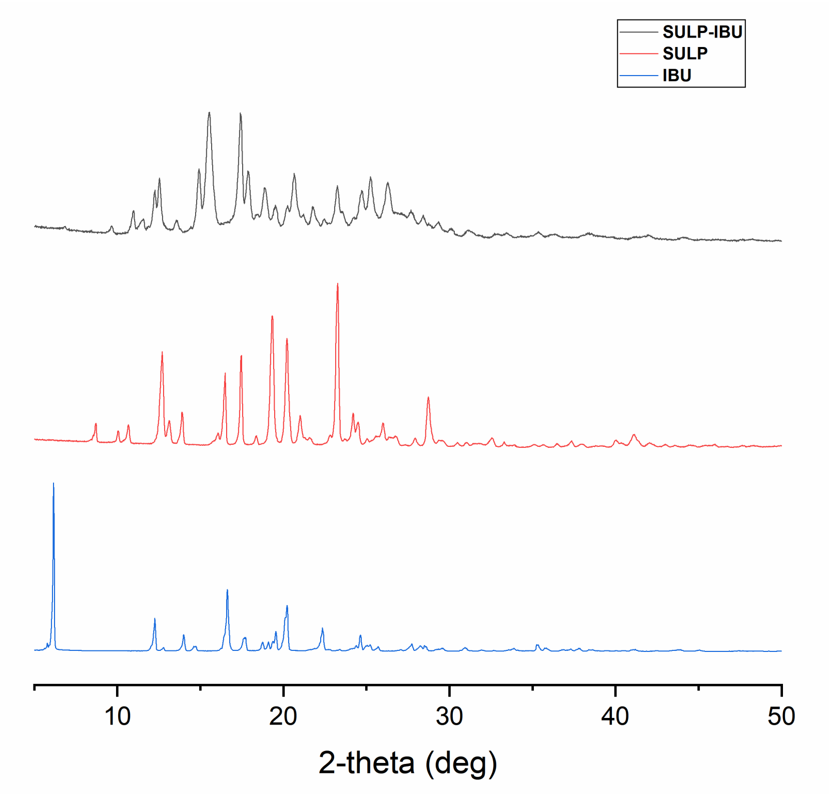

Figure S23. Experimental X-ray powder patterns of **SULP-IBU** (black), SULP (red), IBU (blue).

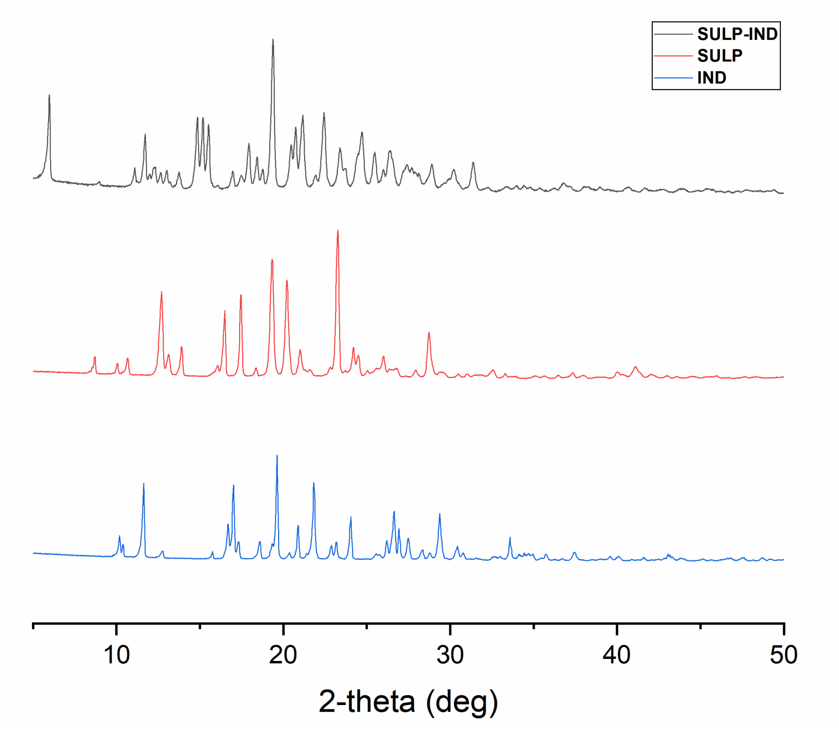

Figure S24. Experimental X-ray powder patterns of **SULP-IND** (black), SULP (red), IND (blue).

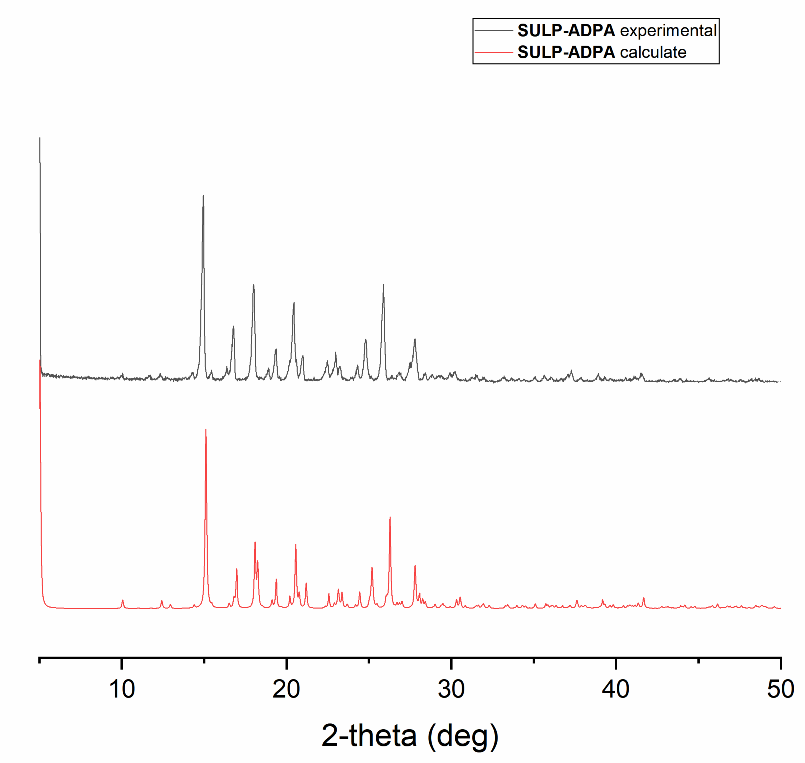

Figure S25. Superimposition of the experimental X-ray powder diffractogram collected on the bulk powder of **SULP-ADPA** and the simulated powder pattern calculated from the structure solved via SCXRD.

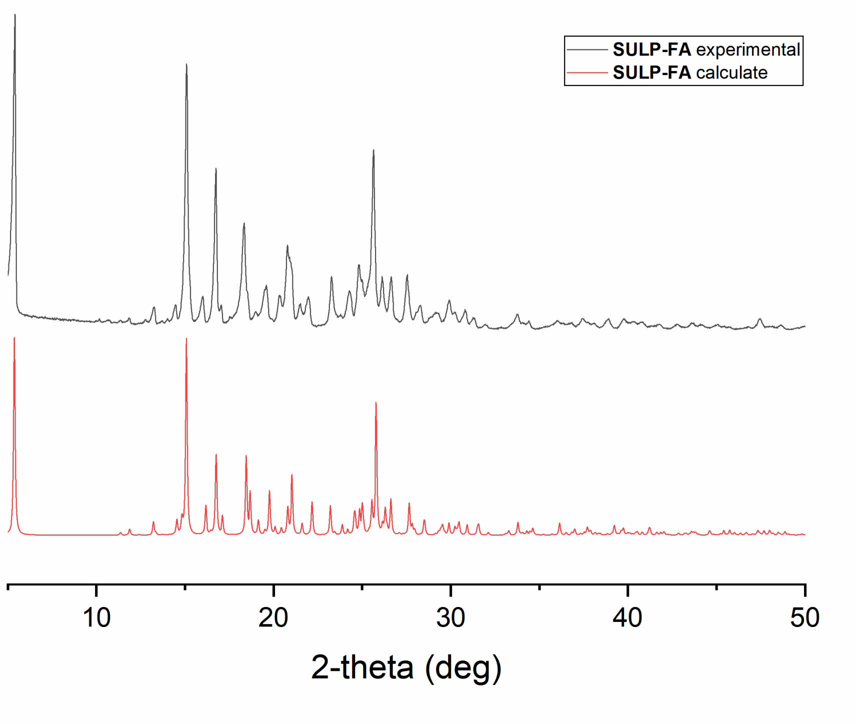

Figure S26. Superimposition of the experimental X-ray powder diffractogram collected on the bulk powder of **SULP-FA** and the simulated powder pattern calculated from the structure solved via SCXRD.

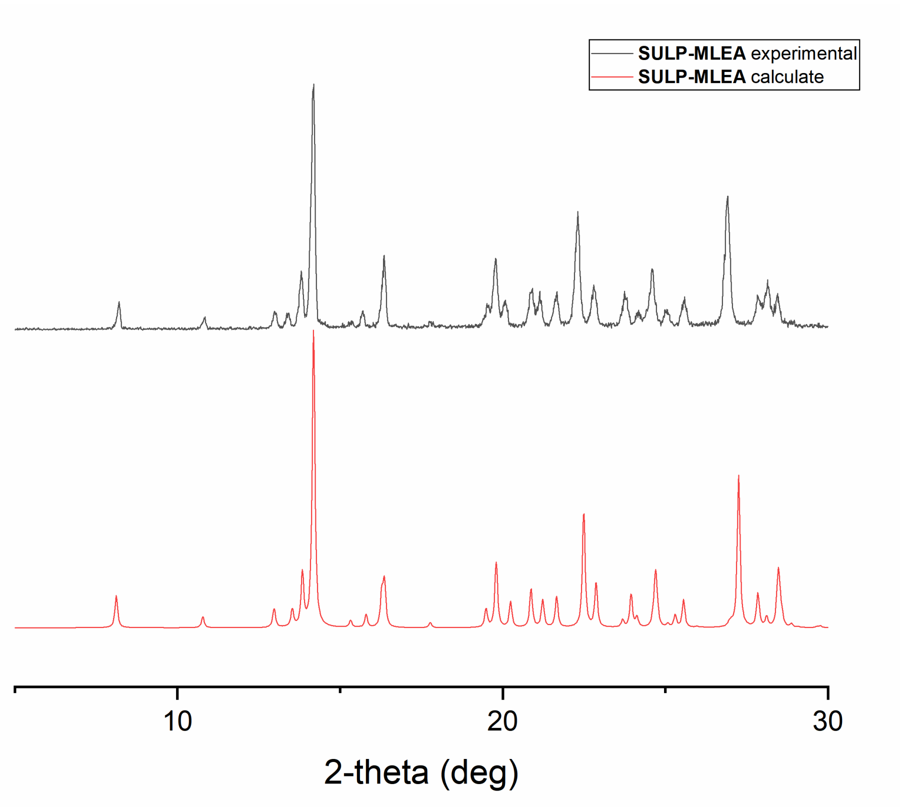

Figure S27. Superimposition of the experimental X-ray powder diffractogram collected on the bulk powder of **SULP-MLEA** and the simulated powder pattern calculated from the structure solved via SCXRD.

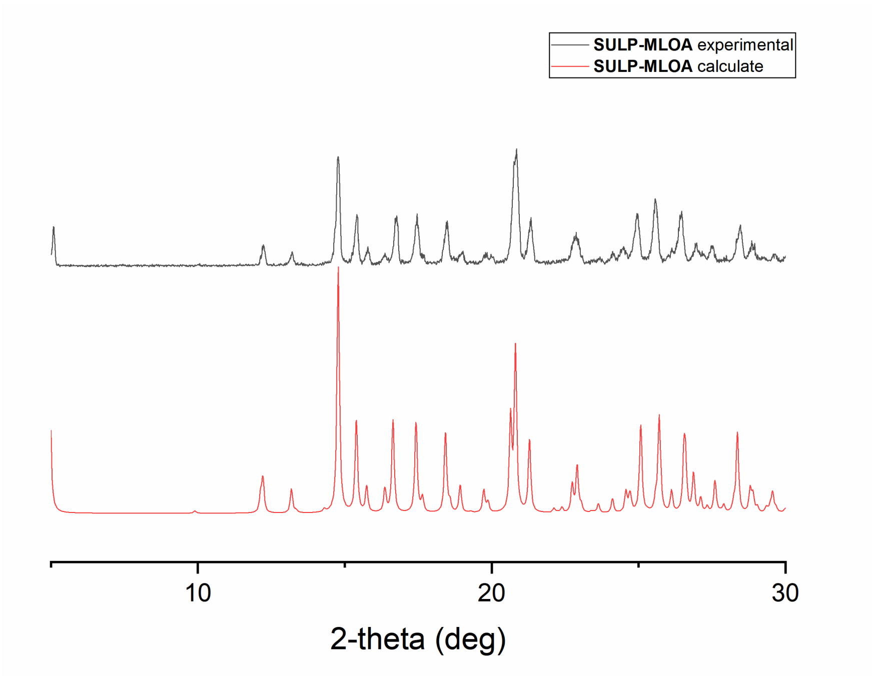

Figure S28. Superimposition of the experimental X-ray powder diffractogram collected on the bulk powder of **SULP-MLOA** and the simulated powder pattern calculated from the structure solved via SCXRD.

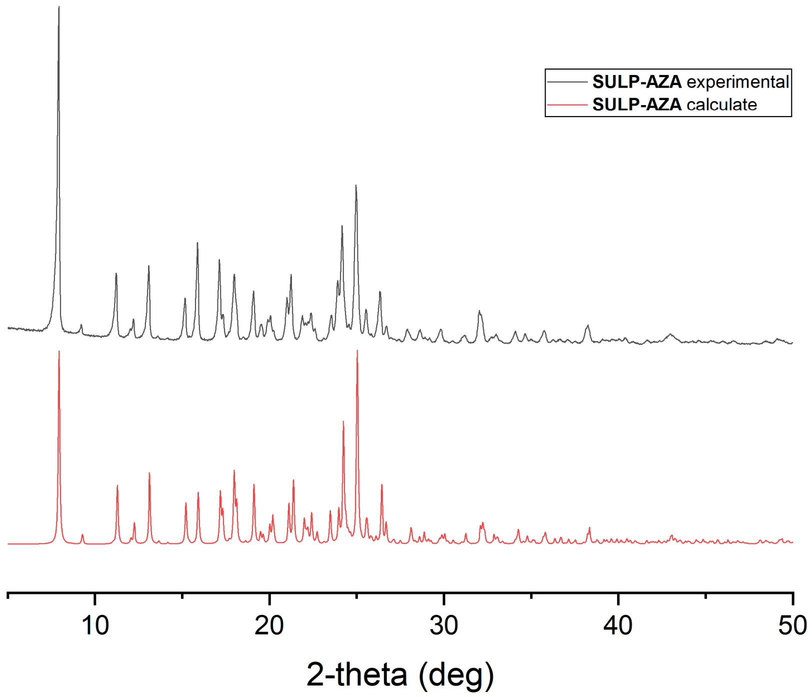

Figure S29. Superimposition of the experimental X-ray powder diffractogram collected on the bulk powder of **SULP-AZA** and the simulated powder pattern calculated from the structure solved via SCXRD.

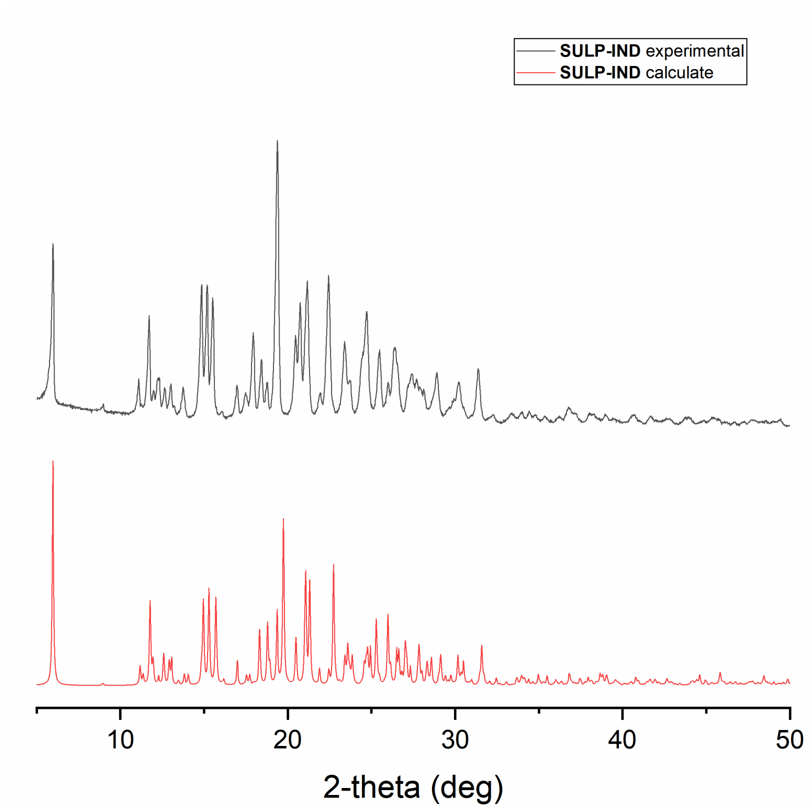

Figure S30. Superimposition of the experimental X-ray powder diffractogram collected on the bulk powder of **SULP-IND** and the simulated powder pattern calculated from the structure solved via SCXRD.

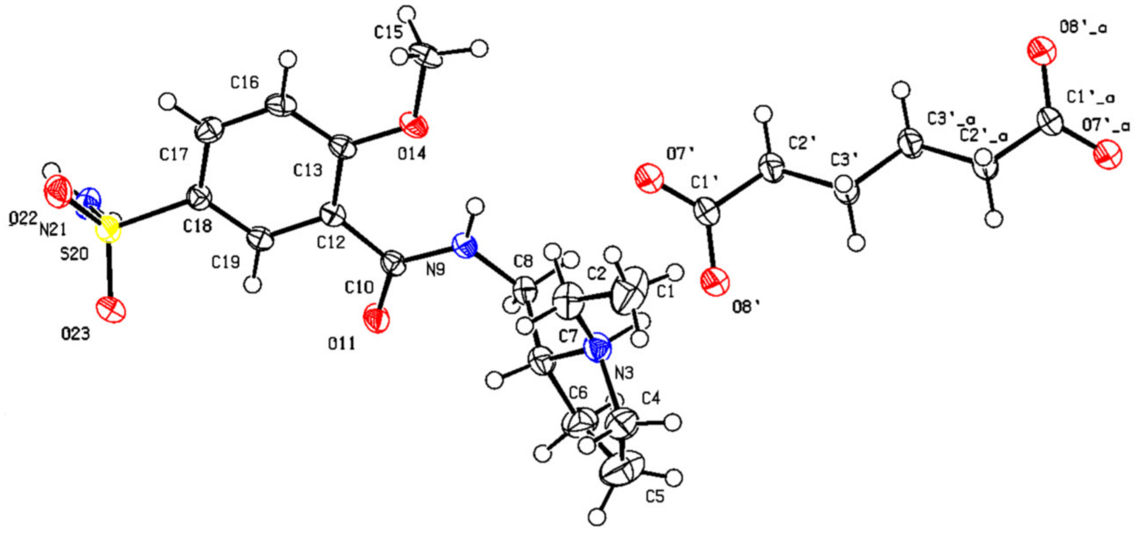

Figure S31. Ortep drawing of **SULP-ADPA**. All non-hydrogen atoms are shown as ellipsoids at the 50% probability level. H atoms (isotropically refined) are reported in ball-and-stick style for the sake of clarity. Colour code: red = O, yellow = S, blue = N, white = C-H. Atoms labelled with "_a" suffix are symmetrically generated.

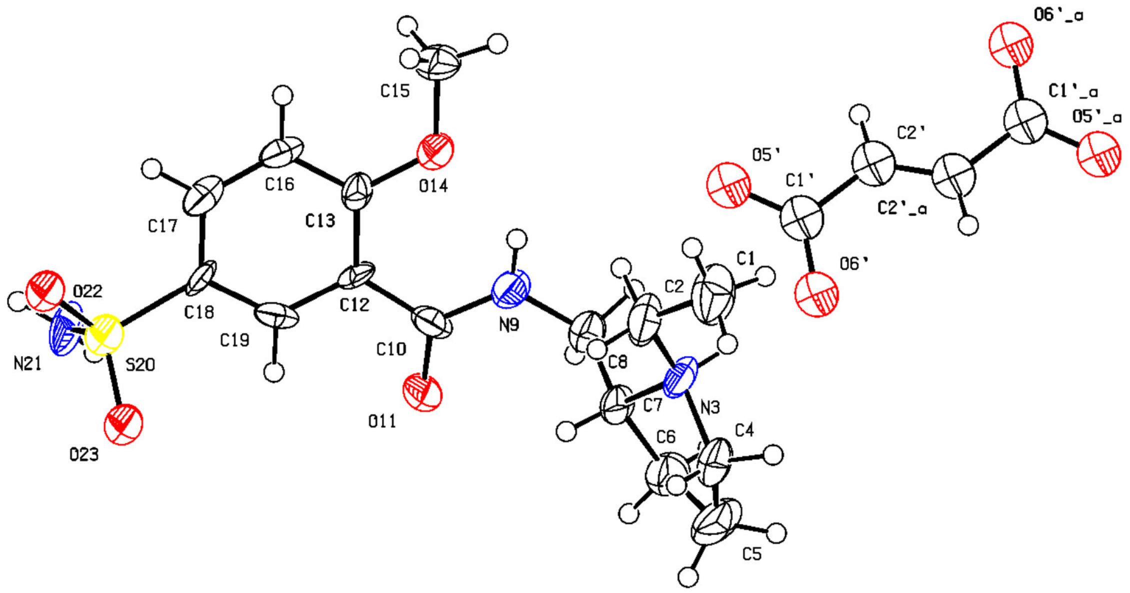

Figure S32. Ortep drawing of **SULP-FA**. All non-hydrogen atoms are shown as ellipsoids at the 50% probability level. H atoms (isotropically refined) are reported in ball-and-stick style for the sake of clarity. Colour code: red = O, yellow = S, blue = N, white = C-H. Atoms labelled with "_a" suffix are symmetrically generated.

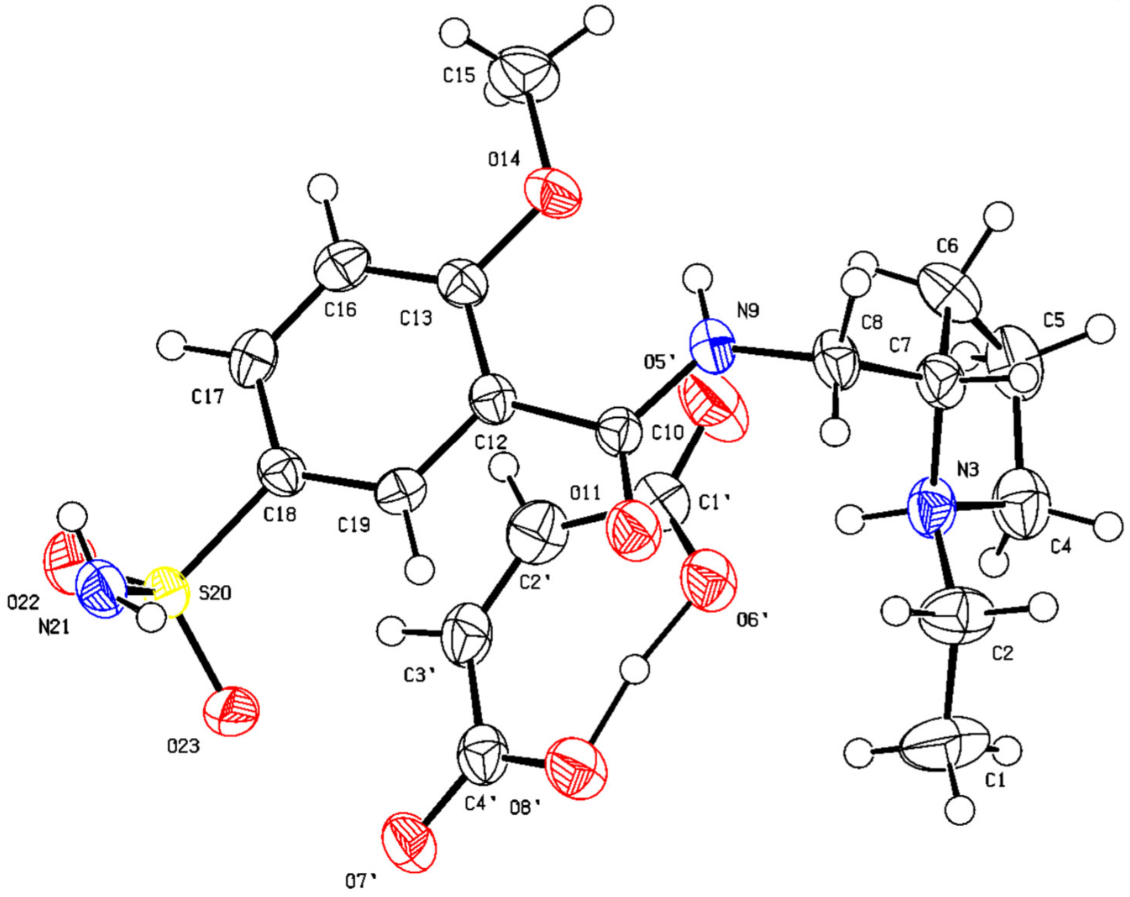

Figure S33. Ortep drawing of **SULP-MLEA**. All non-hydrogen atoms are shown as ellipsoids at the 50% probability level. H atoms (isotropically refined) are reported in ball-and-stick style for the sake of clarity. Colour code: red = O, yellow = S, blue = N, white = C-H.

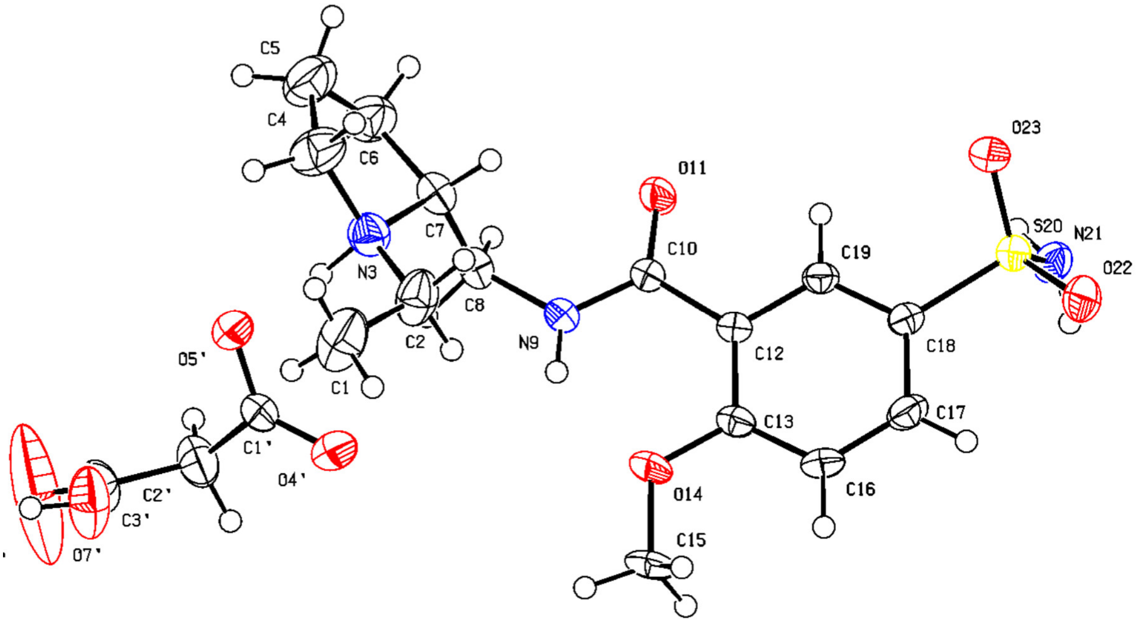

Figure S34. Ortep drawing of **SULP-MLOA**. All non-hydrogen atoms are shown as ellipsoids at the 50% probability level. H atoms (isotropically refined) are reported in ball-and-stick style for the sake of clarity. Colour code: red = O, yellow = S, blue = N, white = C-H.

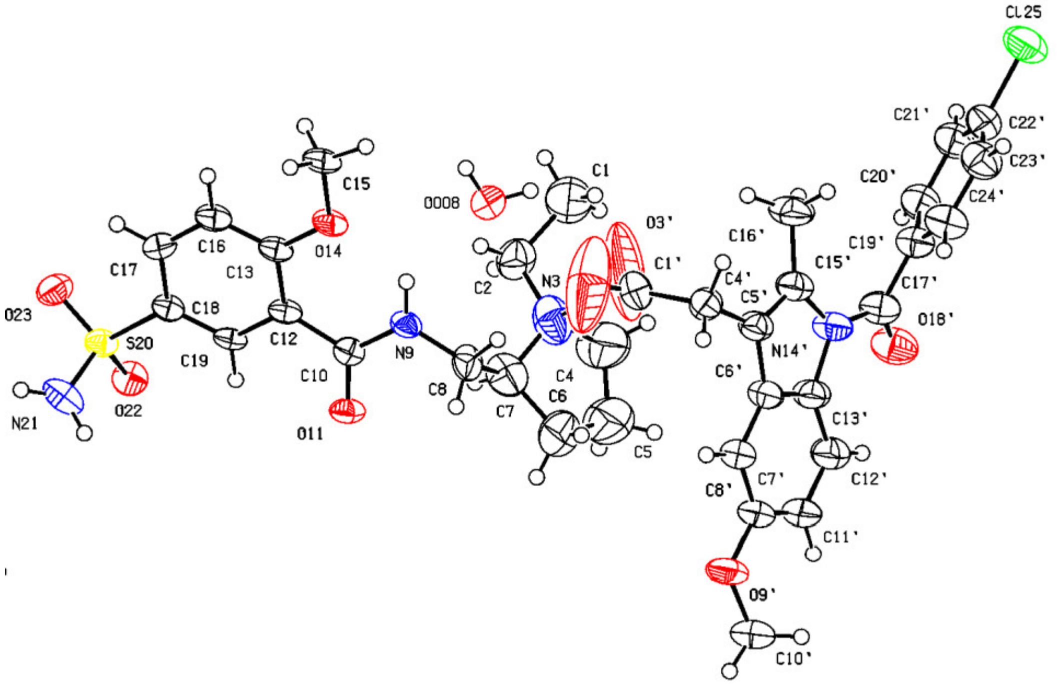

Figure S35. Ortep drawing of **SULP-IND**. All non-hydrogen atoms are shown as ellipsoids at the 50% probability level. H atoms (isotropically refined) are reported in ball-and-stick style for the sake of clarity. Colour code: red = O, yellow = S, blue = N, green = Cl, white = C-H.

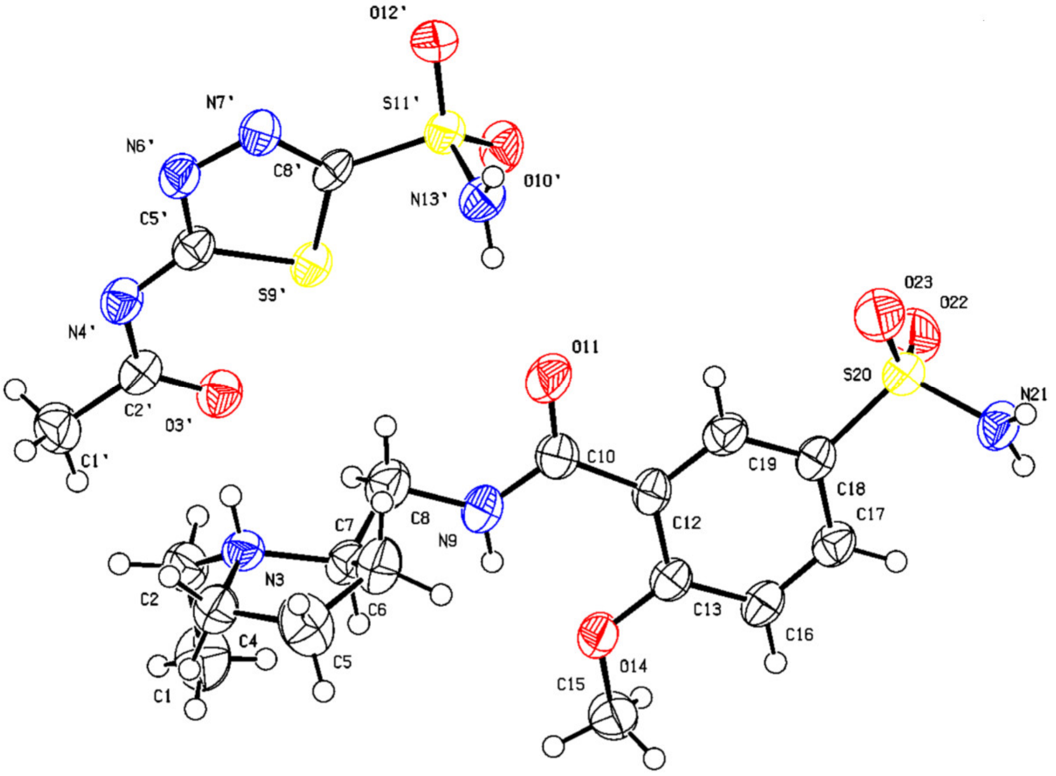

Figure S36. Ortep drawing of **SULP-AZA**. All non-hydrogen atoms are shown as ellipsoids at the 50% probability level. H atoms (isotropically refined) are reported in ball-and-stick style for the sake of clarity. Colour code: red = O, yellow = S, blue = N, white = C-H.

Table S3. H-bonds interactions for all the crystal structures.

	Atoms	Distance (Å)		Atoms	Distance (Å)		Atoms	Distance (Å)
**SULP-ADPA**	N3···O8'	2.64(2)	**SULP-FA**	N3···O6'	2.69(2)	**SULP-IND**	N3···O3'	2.75(2
	N3···O10'	2.64(2)		N3···O8'	2.69(2)		N21···O11	3.00(2)
	N21···O7'	2.78(1)		N21···O5'	2.82(3)		Ow···O2'	2.89(1)
	N21···O9'	2.78(1)		N21···O7'	2.82(3)		Ow···O23	2.93(2)
	N21···O11	2.90(2)		N21···O11	2.98(2)		N9···O14	2.66(3)
	N9···O14	2.67(2)		N21···O14	2.66(3)			
	**Atoms**	**Distance (Å)**		**Atoms**	**Distance (Å)**		**Atoms**	**Distance (Å)**
**SULP-MLEA**	N3···O6'	2.78(3)	**SULP-MLOA**	N3···O5'	2.72(2)	**SULP-AZA**	N3···N7'	2.90(4)
	N21···O5'	2.84(2)		N21···O11	2.91(1)		N21···O10'	2.96(3)
	N21···O7'	2.83(1)		N21···O4'	2.80(1)		N21···O3'	2.95(2)
	N9···O14	2.61(5)		N9···O14	2.67(2)		O11···-N13'	2.86(5)
	O5'···O8'	2.44(2)		O5···O7'	2.58(3)		N4'···N13'	2.97(2)
							N9···O14	2.63(4)

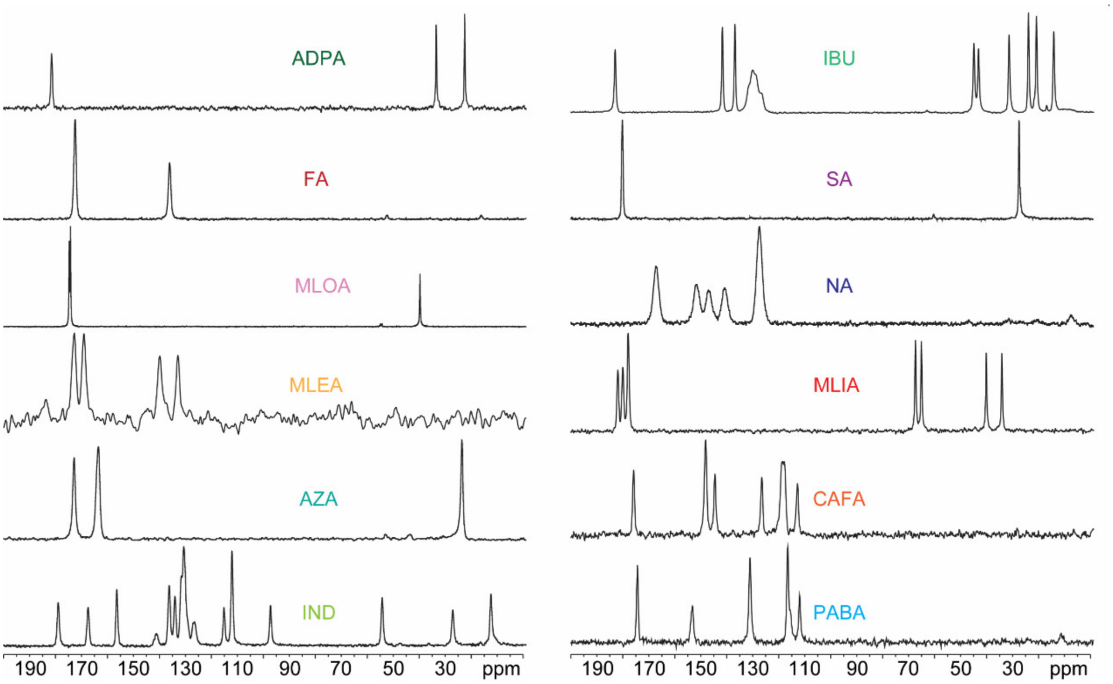

Figure S37. ^{13}C (100.63 MHz) CPMAS spectra of the 12 coformers acquired with a spinning speed of 12 kHz at room temperature.

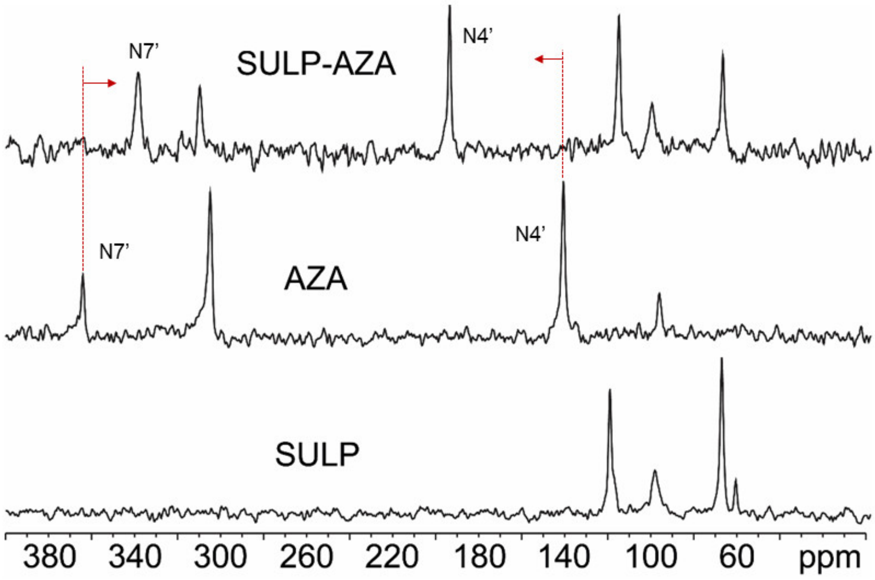

Figure S38. ^{15}N (40.56 MHz) CPMAS spectra of **SULP-AZA**, SULP and AZA acquired with a spinning speed of 8 kHz at room temperature.

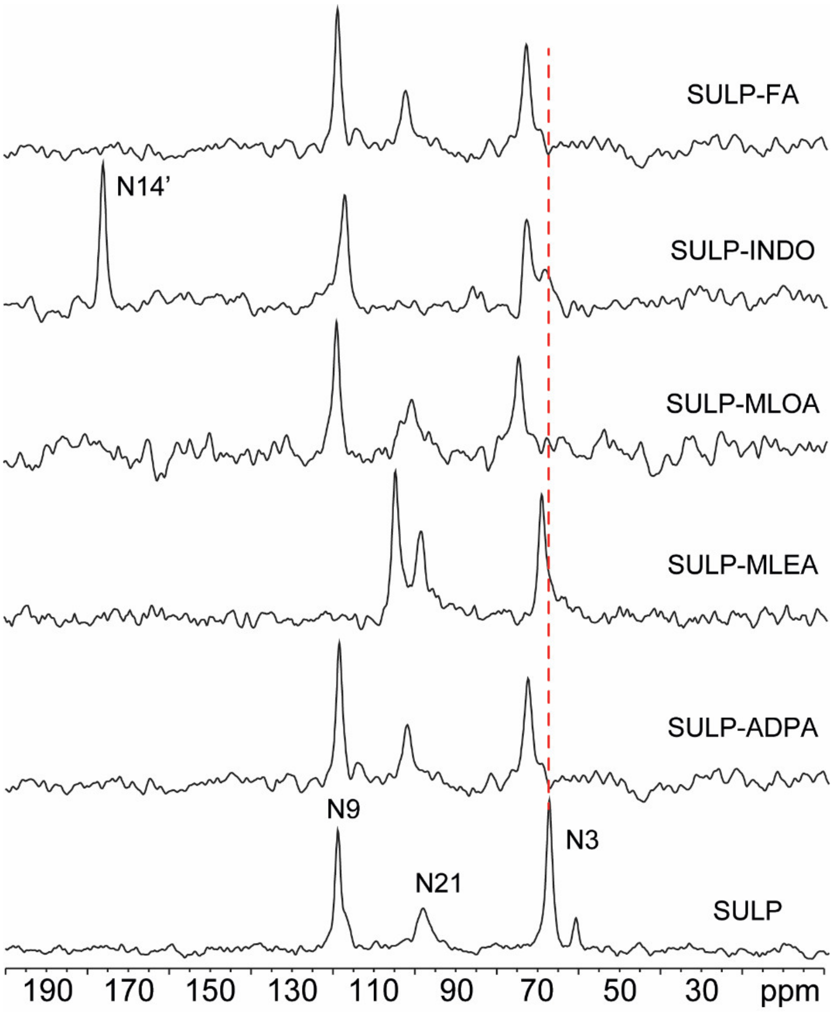

Figure S39. ^{15}N (40.56 MHz) CPMAS spectra of SULP, **SULP-ADPA**, **SULP-MLEA**, **SULP-MLOA**, **SULP-INDO** and **SULP-FA** acquired with a spinning speed of 8 kHz at room temperature.

Table S4. ^{13}C and ^{15}N experimental and computed chemical shifts and relative RMSEs for the SULP-ADPA adduct. Atom numbering refers to Schemes I and II.

SULP-ADPA				
#Atom	^{13}C (ppm)		^{15}N (ppm)	
	Exp	Calc	Exp	Calc
1	11.5	8.6		
1', 6'	182.2	186.5		
2	47.9	46.1		
2', 5'	39.2	37.6		
3			73.6	68.5
3', 4'	28.7	29.1		
4	52.6	51.0		
5	21.0	20.0		
6	28.7	26.6		
7	67.9	66.9		
8	39.2	36.4		
9			119.2	119.4
10	164.6	165.2		
12	120.1	120.7		
13	159.9	162.2		
15	56.5	56.3		
16	111.6	113.4		
17	130.0	130.1		
18	137.5	142.2		
19	130.0	130.2		
21			102.6	107.5
RMSE	2.1		4.1	

Table S5. ^{13}C and ^{15}N experimental and computed chemical shifts and relative RMSEs for the SULP-FA adduct. Atom numbering refers to Schemes I and II.

SULP-FA				
#Atom	^{13}C (ppm)		^{15}N (ppm)	
	Exp	Calc	Exp	Calc
1	12.0	9.8		
1'/4'	172.6	177.9		
2	47.4	45.7		
2'/3'	135.4	138.8		
3			73.6	71.3
4	53.0	49.8		
5	20.1	17.3		
6	28.9	26.2		
7	67.9	67.2		
8	39.4	36.0		
9			119.2	121.4
10	164.8	164.9		
12	120.8	121.1		
13	160.5	161.8		
15	56.7	56.0		
16	112.0	113.2		
17	130.1	130.7		
18	135.4	141.3		
19	130.1	129.4		
21			102.6	102.7
RMSE	2.7		1.9	

Table S6. ^{13}C and ^{15}N experimental and computed chemical shifts and relative RMSEs for the SULP-MLEA adduct. Atom numbering refers to Schemes I and II.

SULP-MLEA				
**#Atom**	**^{13}C (ppm)**		**^{15}N (ppm)**	
	**Exp**	**Calc**	**Exp**	**Calc**
1	10.2	5.6		
1'	168.9	171.7		
2	48.8	48.7		
2'	134.0	137.9		
3			69.2	65.9
3'	136.4	140.9		
4	53.5	51.0		
4'	168.9	171.7		
5	20.0	17.8		
6	25.4	22.9		
7	69.8	70.0		
8	34.1	29.8		
9			104.8	106.5
10	164.5	164.4		
12	121.0	120.1		
13	161.0	162.5		
15	59.0	58.2		
16	114.1	114.0		
17	132.7	133.3		
18	136.4	138.5		
19	130.1	130.3		
21			98.6	100.2
RMSE	2.5		2.4	

Table S7. ^{13}C and ^{15}N experimental and computed chemical shifts and relative RMSEs for the SULP-MLOA adduct. Atom numbering refers to Schemes I and II.

SULP-MLOA				
#Atom	^{13}C (ppm)		^{15}N (ppm)	
	Exp	Calc	Exp	Calc
1	12.1	11.7		
1'	175.5	179.8		
2	48.5	46.1		
2'	45.7	42.7		
3			74.6	70.7
3'	170.5	176.1		
4	53.3	51.0		
5	20.6	18.5		
6	29.2	26.7		
7	68.2	65.8		
8	39.2	37.2		
9			118.8	119.2
10	164.7	164.7		
12	120.4	120.6		
13	160.1	162.1		
15	57.0	56.6		
16	111.6	112.6		
17	130.2	130.3		
18	137.3	141.5		
19	130.2	130.3		
21			100.6	104.1
RMSE	2.5		3.0	

Table S8. ^{13}C and ^{15}N experimental and computed chemical shifts and relative RMSEs for the SULP-IND adduct. Atom numbering refers to Schemes I and II.

SULP-IND				
#Atom	^{13}C (ppm)		^{15}N (ppm)	
	Exp	Calc	Exp	Calc
1	13.2	10.4		
1'	174.9	176.8		
2	48.6	47.2		
3			72.9	64.9
4	53.6	51.1		
4'	33.2	34.0		
5	20.7	17.9		
5'	120.7	121.1		
6	28.9	27.4		
6'	134.6	134.6		
7	66.4	63.7		
7'	105.0	103.2		
8	41.1	38.6		
8'	157.3	159.8		
9			117.0	111.1
10	164.8	164.4		
10'	56.5	55.2		
11'	107.5	105.4		
12	118.9	120.8		
12'	118.1	118.9		
13	160.5	161.7		
13'	132.7	132.7		
14'			175.8	178.5
15	55.8	55.0		
15'	136.3	138.3		
16	111.8	112.1		
16'	16.1	15.9		
17	129.4	129.2		
17'	167.7	169.1		
18	136.3	137.4		
19	128.6	129.0		
19'	134.6	136.7		
20'	130.1	129.4		
21			86.1	97.7
21'	131.7	132.5		
22'	139.4	144.7		
23'	130.1	130.8		
24'	133.7	134.1		
RMSE	1.8		7.8	

Table S9. ^{13}C and ^{15}N experimental and computed chemical shifts and relative RMSEs for the SULP-AZA adduct. Atom numbering refers to Schemes I and II.

SULP-AZA				
#Atom	^{13}C (ppm)		^{15}N (ppm)	
	Exp	Calc	Exp	Calc
1	6.5	1.8		
1'	27.1	24.4		
2	43.1	40.7		
2'	179.5	181.7		
3			68.4	66.6
4	51.4	48.6		
4'			194.8	203.3
5	23.1	21.7		
5'	171.2	173.0		
6	29.0	26.8		
6'			310.3	314.3
7	61.6	62.4		
7'			338.8	332.7
8	39.5	36.4		
8'	162.1	166.6		
9			116.5	119.3
10	159.9	161.3		
12	119.9	120.0		
13	159.9	162.4		
13'			101.3	106.7
15	57.8	58.6		
16	114.5	115.3		
17	129.4	129.9		
18	136.3	140.6		
19	127.3	127.1		
21			101.3	88.5
RMSE	2.5		6.9	
